# Repetitive Elements Contribute to the Diversity and Evolution of Centromeres in the Fungal Genus *Verticillium*

**DOI:** 10.1128/mBio.01714-20

**Published:** 2020-09-08

**Authors:** Michael F. Seidl, H. Martin Kramer, David E. Cook, Gabriel L. Fiorin, Grardy C. M. van den Berg, Luigi Faino, Bart P. H. J. Thomma

**Affiliations:** a Theoretical Biology & Bioinformatics, Utrecht University, Utrecht, the Netherlands; b Laboratory of Phytopathology, Wageningen University, Wageningen, the Netherlands; c Plant Pathology, Kansas State University, Manhattan, Kansas, USA; d Environmental Biology Department, Sapienza Università di Roma, Rome, Italy; e University of Cologne, Institute for Plant Sciences, Cluster of Excellence on Plant Sciences (CEPLAS), Cologne, Germany; Duke University

**Keywords:** centromere, chromosome evolution, heterochromatin, *Verticillium*

## Abstract

Centromeres are chromosomal regions that are crucial for chromosome segregation during mitosis and meiosis, and failed centromere formation can contribute to chromosomal anomalies. Despite this conserved function, centromeres differ significantly between and even within species. Thus far, systematic studies into the organization and evolution of fungal centromeres remain scarce. In this study, we identified the centromeres in each of the 10 species of the fungal genus *Verticillium* and characterized their organization and evolution. Chromatin immunoprecipitation of the centromere-specific histone CenH3 (ChIP-seq) and chromatin conformation capture (Hi-C) followed by high-throughput sequencing identified eight conserved, large (∼150-kb), AT-, and repeat-rich regional centromeres that are embedded in heterochromatin in the plant pathogen Verticillium dahliae. Using Hi-C, we similarly identified repeat-rich centromeres in the other *Verticillium* species. Strikingly, a single degenerated long terminal repeat (LTR) retrotransposon is strongly associated with centromeric regions in some but not all *Verticillium* species. Extensive chromosomal rearrangements occurred during *Verticillium* evolution, of which some could be linked to centromeres, suggesting that centromeres contributed to chromosomal evolution. The size and organization of centromeres differ considerably between species, and centromere size was found to correlate with the genome-wide repeat content. Overall, our study highlights the contribution of repetitive elements to the diversity and rapid evolution of centromeres within the fungal genus *Verticillium*.

## Abstract (precis)

The genus *Verticillium* contains 10 species of plant-associated fungi, some of which are notorious pathogens. *Verticillium* species evolved by frequent chromosomal rearrangements that contribute to genome plasticity. Centromeres are instrumental for separation of chromosomes during mitosis and meiosis, and failed centromere functionality can lead to chromosomal anomalies. Here, we used a combination of experimental techniques to identify and characterize centromeres in each of the *Verticillium* species. Intriguingly, we could strongly associate a single repetitive element to the centromeres of some of the *Verticillium* species. The presence of this element in the centromeres coincides with increased centromere sizes and genome-wide repeat expansions. Collectively, our findings signify a role of repetitive elements in the function, organization, and rapid evolution of centromeres in a set of closely related fungal species.

## INTRODUCTION

Centromeres are crucial for reliable chromosome segregation during mitosis and meiosis. During this process, centromeres direct the assembly of the kinetochore, a multiprotein complex that facilitates attachment of spindle microtubules to chromatids (1–3). Failure in formation or maintenance of centromeres can lead to aneuploidy, i.e., changes in the number of chromosomes within a nucleus, and to chromosomal rearrangements (3–5). While these processes have been often associated with disease development (6), they can also provide genetic diversity that is beneficial for adaptation to novel or changing environments (7, 8). For example, aneuploidy in the budding yeast Saccharomyces cerevisiae can lead to increased fitness under selective conditions, such as the presence of antifungal drugs (9, 10). Thus, centromeric instability can contribute to adaptive genome evolution (11, 12).

Despite their conserved function, centromeres are among the most rapidly evolving genomic regions (13, 14) that are typically defined by their unusual (AT-rich) sequence composition, low gene and high repeat density, and heterochromatic nature (13, 15). Nevertheless, centromeres differ significantly in size, composition, and organization between species (13, 16). Centromeres in S. cerevisiae are only ∼125 nucleotides (nt) long and are bound by a single nucleosome containing the centromere-specific histone 3 variant CenH3 (also called CENP-A or Cse4) (17–20). In contrast to these “point centromeres,” centromeres in many other fungi are more variable and larger and have thus been referred to as “regional centromeres” (15). For instance, in the opportunistically pathogenic yeast Candida albicans, the CenH3-bound 3- to 5-kb-long centromeric DNA regions differ significantly between chromosomes and rapidly diverged from closely related *Candida* species (21–23). Centromeres in the basidiomycete yeasts *Malassezia* are similar in size (3 to 5 kb) but contain a short AT-rich consensus sequence in multiple *Malassezia* species (11). In *Malassezia*, chromosomal rearrangements and karyotype changes are driven by centromeric loss through chromosomal breakage or by inactivation through sequence diversification (11). Chromosomal rearrangements at centromeres have been similarly observed in the yeast Candida parapsilosis, suggesting that centromeres can be fragile and contribute to karyotype evolution (11, 12). CenH3-bound centromeric regions of the basidiomycete yeast Cryptococcus neoformans are relatively large, ranging from 30 to 65 kb, and are rich in long terminal repeat (LTR)-type retrotransposons (16). Centromere sizes differ between *Cryptococcus* species as those lacking RNA interference (RNAi) and DNA methylation have shorter centromeres, associated with the loss of full-length LTR retrotransposons at centromeric regions, suggesting that functional RNAi together with DNA methylation is required for centromere stability (16).

In filamentous fungi, centromeres have been most extensively studied in the saprophyte Neurospora crassa (15). In this species, centromeric regions are considerably larger than in yeasts (on average ∼200 kb) and are characterized by AT-rich sequences that are degenerated remnants of transposable elements and sequence repeats that lack an overall consensus sequence (15, 24, 25). The increased AT content and the degenerated nature of transposable elements in the genome of N. crassa are the result of a process called repeat-induced point mutation (RIP) (15, 26). RIP has been linked to the sexual cycle of ascomycetes and targets repetitive sequences by inducing C-to-T mutations, preferably at CpA dinucleotides (26). The AT-rich centromeric regions are bound by CenH3 and enriched in the heterochromatin-specific histone modification histone 3 trimethylation of lysine 9 (H3K9me3) (25). Additionally, H3K9me3 and cytosine methylation occur at the periphery of the centromeres (25). Alterations in H3K9me3 localization compromise centromeric localization, suggesting that the formation and location of heterochromatin, rather than the DNA sequence itself, are essential for function and localization of centromeres in N. crassa (15, 25). However, heterochromatin is not a hallmark for centromeres in all filamentous fungi. Centromeres in the fungal wheat pathogen Zymoseptoria tritici are shorter (∼10 kb) and AT-poor, and their presence does not correlate with transposable elements nor with heterochromatin-specific histone modifications such as H3K9me3 or histone 3 trimethylation of lysine 27 (H3K27me3) (27). Thus, even though centromeric function is highly conserved, fungal centromeres differ considerably in size, sequence composition, and organization.

Knowledge on centromeres has been impaired by their repetitive nature, which hampers their assembly and subsequent analyses (15, 28). However, recent advances in long-read sequencing technologies enable studies of the constitution and evolution of centromeres (11, 16, 29–31). By using long-read sequencing technologies in combination with optical mapping, we previously generated gapless genome assemblies of two strains of the fungal plant pathogen Verticillium dahliae (32). The genome of V. dahliae is characterized by lineage-specific (LS) regions (7, 8, 33–35) that are hypervariable between V. dahliae strains and that contain genes with crucial roles in virulence and host adaptation (7, 8, 33, 35). LS regions evolved by extensive chromosomal rearrangements such as translocations, inversions, duplications, or deletions, that are mediated by erroneous double-strand repair pathways, often involving repetitive elements (8). Repetitive elements within the LS regions display a distinct chromatin state compared with other repetitive regions (36). The *Verticillium* genus consists of 10 species that are all soilborne and presumed asexual but have different lifestyles (37). Nine of these species are haploid, while the species Verticillium longisporum is an allodiploid hybrid between a strain that is closely related to V. dahliae and an unknown *Verticillium* species (37–39). During the evolution of the different *Verticillium* species, frequent chromosomal rearrangements occurred (8, 35, 40), and regions with characteristics similar to LS regions have been identified in other *Verticillium* species as well (33). Centromeres have been thought to facilitate chromosomal rearrangements and contribute to karyotype evolution (11, 12, 41), and thus deeper knowledge of centromeres might help in understanding mechanisms that drive chromosomal rearrangements in *Verticillium* genome evolution. Facilitated by the availability of V. dahliae high-quality genome assemblies and of all other *Verticillium* species (32, 33, 40, 42), we here sought to identify and study the constitution and evolution of centromeres in the *Verticillium* genus and to elucidate their impact on chromosome evolution.

## RESULTS

### CenH3 binding identifies large regional centromeres in Verticillium dahliae.

**FIG 1:**
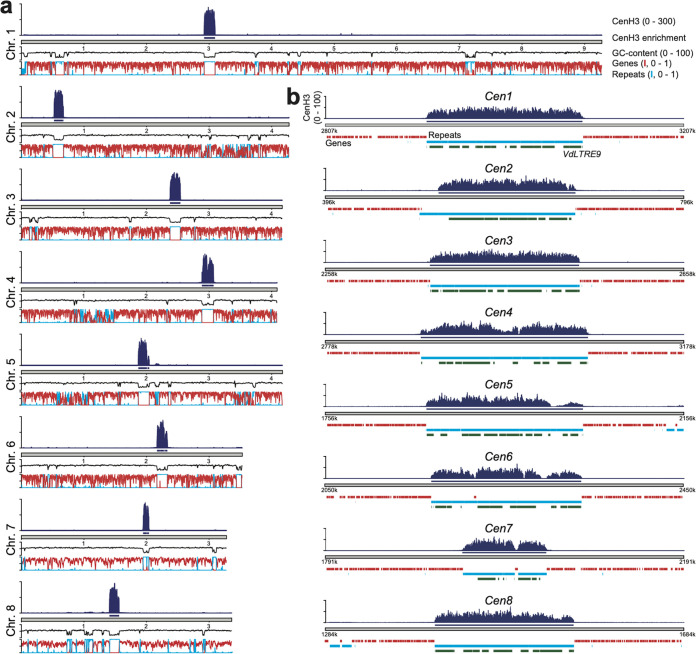
CenH3 binding defines centromeres in Verticillium dahliae strain JR2. (a) Schematic overview of the chromosomes of V. dahliae strain JR2 showing the normalized CenH3 ChIP-seq read coverage (RPGC normalization in 1-kb bins with 3-kb smoothening), CenH3 enriched regions, GC content, gene density (red line), and repeat density (blue line). (b) Magnification of a 400-kb region containing the centromere is shown for each of the eight chromosomes of V. dahliae strain JR2 (*Cen1* to -*8*) depicting the CenH3 ChIP-seq read coverage (RPGC normalization in 10-bp bins with a 30-bp smoothening) and enrichment, as well as the presence of genes (red) and repetitive elements (blue). Regions carrying the centromere-specific long terminal repeat element *VdLTRE9* are highlighted in green.

Centromeres differ significantly between fungi, but most centromeres are functionally defined by nucleosomes containing CenH3 (1). To identify centromeres in V. dahliae strain JR2 by chromatin immunoprecipitation followed by high-throughput sequencing (ChIP-seq), we first identified the V. dahliae CenH3 ortholog (see Fig. S1a in the supplemental material) and generated transformants with N-terminally FLAG-tagged CenH3 (Table S1). To this end, the coding sequence for the FLAG-tagged CenH3 was inserted in the locus behind the native *CenH3* promoter (Fig. S1b and c). We subsequently used anti-FLAG antibodies to purify FLAG-tagged CenH3-containing nucleosomes from two V. dahliae transformants (Table S1a) and sequenced the nucleosome-associated genomic DNA. Mapping of the sequencing reads to the V. dahliae strain JR2 genome assembly identified a single CenH3-enriched region per chromosome (Fig. 1a; Fig. S1d and e), while mapping of the sequencing reads derived from the wild-type (WT) strain did not reveal any CenH3-enriched region (Fig. S1d and e). The CenH3-enriched regions, designated *Cen1* to *Cen8*, range between ∼94 and ∼187 kb in size (Fig. 1a; Table 1). To corroborate these centromere sizes, we assessed centromere locations based on a previously generated optical map (32, 35) revealing no significant size differences (Fig. S1e). Thus, we conclude that CenH3 binding defines large regional centromeres in V. dahliae strain JR2.

**TABLE 1:** Genome characteristics of the centromeres of Verticillium dahliae strain JR2^*e*^

Chr.	Locus	CenH3		AT-rich, position (kb)^*b*^	AT content (%)^*c*^		Repetitive elements	
		Position (bp)^*a*^	Length (bp)		Chr.	Cen.	No. of repeats (%)^*d*^	No. of *VdLTRE9* (%)^*d*^
1	*CEN1*	2920143–3094179	174,037	2919–3094	45.7	77.1	50 (99.8)	27 (70.4)
2	*CEN2*	520698–672281	151,584	516–672	46.3	77.8	43 (99.7)	26 (83.0)
3	*CEN3*	2374294–2541026	166,733	2375–2542	45.8	77.3	47 (99.8)	31 (80.5)
4	*CEN4*	2884316–3071412	187,097	2885–3072	46.2	75.4	54 (99.5)	24 (53.8)
5	*CEN5*	1868317–2043260	174,944	1868–2044	46.7	73.9	58 (99.5)	25 (63.1)
6	*CEN6*	2166972–2333060	166,089	2167–2334	46.4	75.2	48 (100)	31 (62.6)
7	*CEN7*	1944367–2038091	93,725	1945–2038	44.7	76.5	32 (95.8)	14 (47.8)
8	*CEN8*	1406398–1561664	155,267	1406–1562	47.7	77.0	37 (100)	26 (73.9)

a Position of CenH3-enriched domains; enriched domains within 10 kb have been merged.

b Position of AT-rich domains; AT-rich domains within 20 kb have been merged.

c Average AT content of 1-kb windows of the entire chromosome and the AT-rich domain.

d Percentage of centromeric region covered.

e Abbreviations: Chr., chromosome; Cen., centromere.

10.1128/mBio.01714-20.1 FIG S1 (a) Phylogenetic analyses of the canonical H3 and the centromere-specific CenH3 in Verticillium dahliae (strain JR2) and other fungal genomes. (b and c) Transformation of the coding sequence of N-terminally FLAG-tagged CenH3 directed by its native promoter at the *CenH3* locus in Verticillium dahliae strain JR2. (b) Correct homologous recombination and replacement at the *CenH3* locus were verified by PCR amplification and assessed using PCR. (c) Correct translation of the recombinant protein was assessed using Western blot analyses with anti-FLAG antibody. (d) Sequencing read coverage (RPGC normalization in 1-kb bins with 3-kb smoothening) from ChIP-seq experiments using FLAG tag antibodies on two independent transformants of Verticillium dahliae strain JR2 that express FLAG-tagged CenH3 and the wild-type strain are mapped to the eight chromosomes of V. dahliae strain JR2 (32). Gene (red) and repeat (blue) densities are shown below each chromosome. (e) Principal-component analysis of the four FLAG tag ChIP-seq samples (two wild type and two FLAG-CenH3). (f) Comparison of the centromeric regions with the identified centromeres highlighted as blue block in the genome assembly of Verticillium dahliae strain JR2 with a previously generated optical map (35). Vertical lines display corresponding (*in silico*) restriction sites and their alignment. Download FIG S1, JPG file, 1.1 MB. Copyright © 2020 Seidl et al. 2020 Seidl et al. This content is distributed under the terms of the Creative Commons Attribution 4.0 International license.

10.1128/mBio.01714-20.10 TABLE S1 (a) Overview of the different *Verticillium* sequencing libraries used in this study. (b) Position of the individual centromeric regions inferred by Hi-C interchromosomal interaction frequencies and the overlap (in kb) with CenH3-enriched regions and the centromere-associated *VdLTRE9* in Verticillium dahliae JR2, VdLs17, and CQ2. (c) Overview of the different *Verticillium* genomes assembled using Hi-C interactions. (d) Position, length, and number of assembly gaps (N’s) of the individual centromeric regions inferred by Hi-C interchromosomal interaction in *Verticillium nonalfalfae* (T2), *Verticillium alfalfae* (PD683), the allodiploid *Verticillium longisporum* (PD589), *Verticillium nubilum* (397), *Verticillium albo-atrum* (PD747), *Verticillium zaregamsianum* (PD739), *Verticillium tricorpus* (PD593), *Verticillium klebahnii* (PD401), and *Verticillium isaacii* (PD618). (e) The number of *de novo* repeat consensus sequences identified within and outside centromeric regions in the *Verticillium* species. Only consensus elements with >5 matches in centromeric regions are displayed. Note that the consensus names between species/strains are not comparable. (f) The presence of RNAi components (argonaute, dicer, RNA-dependent RNA polymerase; protein sequences derived from the work of Jesenicnik et al. [75]) in the genome assemblies of the 10 different *Verticillium* species was assessed using manual BLAST (tBLASTn v2.9.0+; default settings) (76, 77) and exonerate (v2.2.0; default settings) (78) searches. (g) The primers used for cloning the CenH3 FLAG tag in Verticillium dahliae strain JR2. Download Table S1, XLSX file, 0.04 MB. Copyright © 2020 Seidl et al. 2020 Seidl et al. This content is distributed under the terms of the Creative Commons Attribution 4.0 International license.

### Centromeres in Verticillium dahliae are repeat rich and embedded in heterochromatin.

Centromeres are often characterized by increased AT content, increased repeat density, and depletion of protein-coding genes (13, 15, 29). To characterize the centromeres in V. dahliae strain JR2, we queried the eight chromosomes for the presence of large AT-rich, gene-sparse, and repeat-rich regions. Seven of the eight chromosomes contain only a single large (>93 kb; average size, ∼150 kb) AT-rich region (∼74 to 78% versus ∼46% genome-wide), nearly completely devoid of protein-coding genes and enriched for repetitive sequences, that overlaps the regions defined by CenH3 binding (Fig. 1a; Table 1). In contrast, chromosome 1 contains three regions with these characteristics (Fig. 1a; Table 1). However, only one of these overlaps the centromeric regions defined by CenH3 binding (Fig. 1).

**FIG 2:**
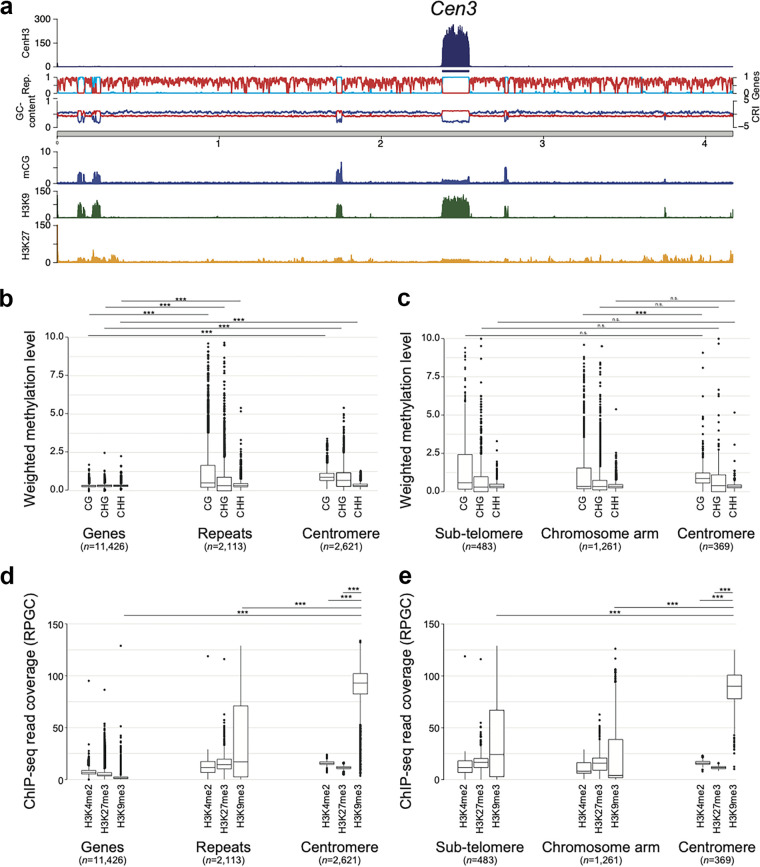
Centromeres in Verticillium dahliae strain JR2 are embedded in heterochromatin. (a) Schematic overview of chromosome 3 of V. dahliae strain JR2, exemplifying the distribution of heterochromatin-associated chromatin modifications (mC, H3K9me3, and H3K27me3) in relation to the centromeres. The different lanes display the FLAG-CenH3 ChIP-seq read coverage (RPGC normalization in 1-kb bins with 3-kb smoothening), the FLAG-CenH3 enriched regions, the repeat and gene density (light blue and red, respectively), the GC content (blue), the CRI (red) as well as the weighted cytosine methylation (all summarized in 5-kb windows with 500-bp slide), and the normalized H3K9me3 and H3K27me3 ChIP-seq read coverage (RPGC normalization in 1-kb bins with 3-kb smoothening). The schematic overview of all chromosomes is shown in Fig. S2. (b) Boxplots of weighted DNA methylation levels per genomic context (CG, CHG, or CHH) are summarized over genes, repetitive elements, or 5-kb genomic windows (500-bp slide) overlapping the centromeric regions. (c) Weighted DNA methylation levels per genomic context (CG, CHG, or CHH) are summarized over repetitive elements that have been split based on their genomic location: subtelomeres (within the first or last 10% of the chromosome), centromeres, or the remainder of the chromosome arm. (d) ChIP-seq read coverage (RPGC normalized; see panel a for H3K4me2, H3K27m3, and H3K9me3) is summarized over genes, repetitive elements, or 5-kb windows (500-bp slide) overlapping the centromeric regions. (e) ChIP-seq read coverage (RPGC normalized; see panel a for H3K4me2, H3K27m3, and H3K9me3) is summarized over repetitive elements that have been split based on their genomic location: subtelomeres (within the first or last 10% of the chromosome), centromeres, or the remainder of the chromosomal arm. Statistical differences for the indicated comparisons were calculated using the one-sided nonparametric Mann-Whitney test; *P* values <0.001, ***; n.s., not significant.

Elevated AT levels in repeat-rich regions are caused by RIP mutations in some filamentous fungi (15, 25, 26, 43). Due to its presumably asexual nature (7), the occurrence of RIP in V. dahliae is controversial (8, 44, 45), although signatures of RIP have previously been reported in a subset of repeat-rich regions (36). We assessed the occurrence of RIP signatures in centromeres using the composite RIP index (CRI) (46), which considers C-to-T mutations in the CpA context. Intriguingly, genomic regions located at centromeres display significantly higher CRI values than other genomic regions (e.g., genes or repetitive elements) (Fig. 2a; Fig. S2 and S3a), and thus, RIP signatures at repetitive elements located at centromeres likely contribute to the high AT levels.

10.1128/mBio.01714-20.2 FIG S2 Schematic overview of the eight chromosomes of Verticillium dahliae strain JR2 displaying different heterochromatin-associated chromatin modifications (mC, H3K9me3, and H3K27me3) in relation to the centromeres. The different lanes display the FLAG-CenH3 ChIP-seq read coverage (RPGC normalization in 1-kb bins with 3-kb smoothening), the repeat density, the GC content, the CRI as well as the weighted cytosine methylation (all summarized in 5-kb windows with 500-bp slide), and the normalized H3K9me3 and H3K27me3 ChIP-seq read coverage (RPGC normalization in 1-kb bins with 3-kb smoothening). Download FIG S2, JPG file, 0.8 MB. Copyright © 2020 Seidl et al. 2020 Seidl et al. This content is distributed under the terms of the Creative Commons Attribution 4.0 International license.

10.1128/mBio.01714-20.3 FIG S3 (a) Boxplot displaying the composite RIP index (CRI) of C to T in CA recorded in genomic windows (5 kb, 500-bp slide), per gene, per annotated repeat, and per window overlapping the CenH3-enriched centromeres. Statistical differences for the indicated comparisons were calculated using the one-sided nonparametric Mann-Whitney test; *P* values <0.001, ***. (b) Summary of H3K4me2 (green), H3K9me3 (red), and H3K27me3 (orange) normalized ChIP-seq read coverage (RPGC normalization in 1-kb bins and 3-kb smoothening) in genomic bins (2.5%) across the chromosomal arms of the eight chromosomes of Verticillium dahliae strain JR2 (divided into 2.5% bins) and the centromeric regions (divided into 10% bins). The dots indicate the average ChIP-seq coverage, and the whiskers indicate ±1.5 times the interquartile range. (c to e) Boxplots displaying the weighted methylation levels (CG context) (c), the composite RIP index (d), and the expression in potato dextrose broth (PDB) growth medium (e) (counts per million) for repetitive elements belonging to 10 repeat families identified in the eight centromeres in Verticillium dahliae JR2. (f) The distribution of different repeat subfamilies in centromeres (*Cen*) and across the genome (non-*Cen*) and separated by full-length and fragmented elements. Download FIG S3, JPG file, 0.6 MB. Copyright © 2020 Seidl et al. 2020 Seidl et al. This content is distributed under the terms of the Creative Commons Attribution 4.0 International license.

In most filamentous fungi and oomycetes, AT- and repeat-rich centromeres are embedded in heterochromatin that is characterized by methylated DNA and by particular histone modifications (H3K9me3 and H3K27me3) (13, 15, 16, 25, 30, 46). We recently determined chromatin states in the genome of V. dahliae strain JR2 and revealed that repetitive sequences outside the LS regions display characteristics of heterochromatin (36). To define centromeric chromatin states, we used previously generated bisulfite sequencing data to monitor DNA methylation (mC) and ChIP-seq data to determine the distribution of the heterochromatic marks H3K9me3 and H3K27me3 (36). To also determine the distribution of euchromatin, we performed ChIP-seq with an antibody against the euchromatic mark dimethylation of lysine 4 of histone H3 (H3K4me2). We observed overall low genome-wide DNA methylation levels (36) (Fig. 2a; Fig. S2), similar to the previously reported levels for Aspergillus flavus (47) and lower than for N. crassa (48). Nevertheless, repetitive elements and centromeres show significantly higher DNA methylation levels in all contexts compared with genes (Fig. 2b). Methylation (in CG context) at repetitive elements at centromeres is significantly higher than at repeats located along the chromosomal arm, but not at subtelomeric regions (Fig. 2c), and more methylation at centromeres correlates with increased CRI (Fig. 2a; Fig. S2 and S3a). DNA methylation colocalizes with H3K9me3 at repeat-rich regions (36) (Fig. 2a; Fig. S2). H3K9me3 occurs predominantly at repetitive elements localized at subtelomeres and centromeres (Fig. 2d and e; Fig. S2 and S3b). In comparison, H3K4me2 and H3K27me3 are largely absent from centromeres (Fig. 2d and e; Fig. S3b). Collectively, these observations indicate that centromeres of V. dahliae display typical characteristics of constitutive heterochromatin.

### A single repeat associates with centromeres of Verticillium dahliae strain JR2.

**FIG 3:**
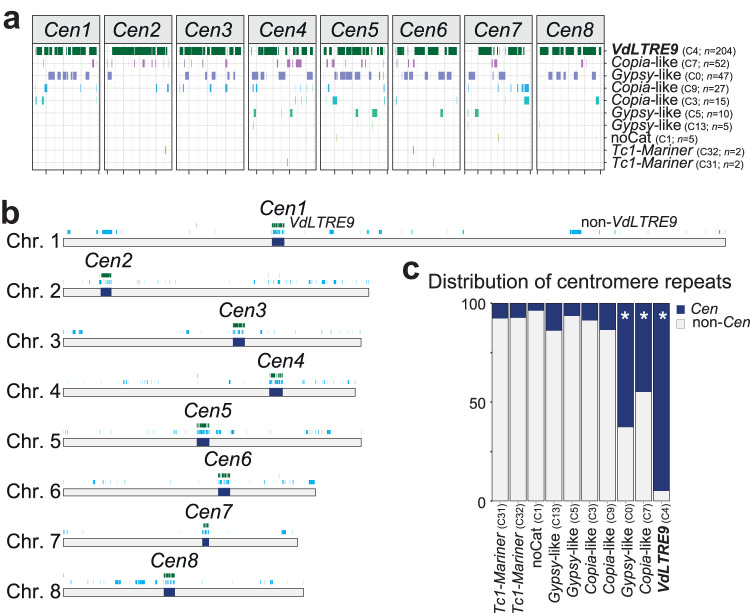
A single repeat family associates with centromeres in Verticillium dahliae strain JR2. (a) The presence of different repeat subfamilies is shown across the eight centromeres (*Cen1* to -*8*), and the number of occurrences for each subfamily within the centromeres is indicated. The individual centromeres in the diagram are shown in equal scale. (b) Genome-wide distribution of the 10 repeat subfamilies occurring within the eight centromeres (*Cen1* to -*8*; dark blue); the location of *VdLTRE9* is shown in green, and the location of elements belonging to the other nine repeat subfamilies (from panel a) is shown in light blue. (c) The distribution of different repeat subfamilies in centromeres (*Cen*; dark blue) and across the genome (non-*Cen*; light gray). The enrichment of specific subfamilies at centromeres was assessed using a one-sided Fisher exact test. Significant enrichment (multiple-testing corrected *P* value < 0.01) is denoted with an asterisk.

Centromere identity and function are typically defined by CenH3 binding and not by specific DNA sequences, although various types of repetitive sequences, such as transposable elements, are commonly observed in centromeres of plants, animals, and fungi (13, 15, 49, 50). Unsurprisingly, CenH3-bound centromeres are repeat rich in V. dahliae (Fig. 1). A detailed analysis of the eight centromeres revealed a nearly complete (>96%) composition of repetitive elements belonging to only 10 different repeat subfamilies (Fig. 1b, Fig. 3a, and Table 1), of which the majority shows similarity to LTR retrotransposons of the *Gypsy*- and *Copia*-like families (Fig. 3a). These elements show signs of RIP, are highly methylated and nontranscribed (Fig. S3c to e), and thus are likely inactive. Interestingly, a single LTR retrotransposon subfamily, previously designated *VdLTRE9* (8, 32), covers on average ∼70% of the DNA sequences at the eight centromeres, ranging from 48% in *Cen7* to 83% in *Cen2* (Fig. 3a; Table 1). We scanned the genome for the localization of the 10 repeat subfamilies (Fig. 3). Intriguingly, although it is one of the most abundant repeats in the genome with 215 complete or partial matches, *VdLTRE9* is associated with centromeres as 95% of the copies (204 out of 215; one-sided Fisher’s exact test; multiple-testing corrected *P* value 3e−106) occur at the eight centromeres (Fig. 3b and c). The remaining 11 *VdLTRE9* copies (5%) occur outside the CenH3-rich centromeres, yet five out of 11 copies are localized within 50 kb of the centromeric regions (Fig. 3b and c). The nine other repeat subfamilies have additional matches that are located outside the centromeres (Fig. 1a and Fig. 3b and c), and only two of these repeats are significantly enriched and consistently present in all eight centromeres; 63% and 45% of the matches of these two subfamilies occur at the centromeres (Fig. 3c). Repeats at centromeres are often fragmented, and most copies, with the exception of the Tc1/mariner-like elements, are similarly fragmented when located outside the centromeres (Fig. S3f), indicating extensive degeneration of repetitive elements in V. dahliae. Collectively, these findings suggest that only the presence of *VdLTRE9* is strongly associated with centromeres in V. dahliae strain JR2.

*VdLTRE9* displays similarity to LTR retrotransposons. The consensus sequence of *VdLTRE9* is ∼7.3 kb long (the two LTR sequences are each ∼200 bp long), and the individual matches share a high degree of sequence identity (∼86%). Sequence similarity-based transposable element classifications using PASTEC (51) indicate that the consensus sequence displays remote similarity to *Gypsy*-like retrotransposons. Only ∼25% of the *VdLTRE9* matches in the genome cover the entire (>97.5%) consensus sequence, but many of these are still fragmented as they occur as discontinuous copies. Furthermore, the *VdLTRE9* consensus sequence is AT rich (∼75% AT), which may be caused by RIP (Fig. S3d), indicating that *VdLTRE9*, similar to other repeats in V. dahliae, has significantly degenerated.

### *VdLTRE9* as hallmark of Verticillium dahliae centromeres.

**FIG 4:**
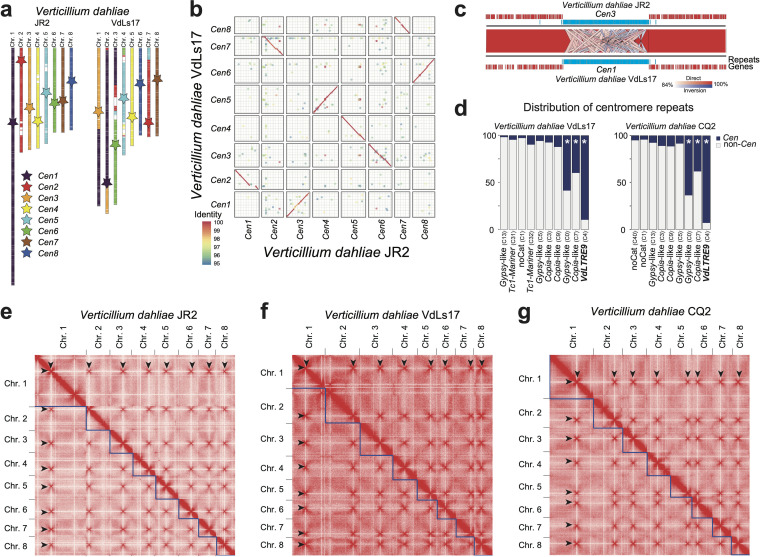
Hi-C contact maps identify *VdLTRE9* as a hallmark of centromeres in Verticillium dahliae. (a) Synteny analyses of the eight chromosomes of V. dahliae strains JR2 and VdLs17. Schematic overview of the eight chromosomes of V. dahliae strain JR2 (left) and the corresponding syntenic regions in V. dahliae strain VdLs17 (right). Approximate locations of centromeres are indicated by stars, and syntenic centromeres of V. dahliae strain VdLs17 are colored according to *Cen1* to -*8* of V. dahliae strain JR2. (b) Sequence alignment of the centromeric regions ±20 kb in V. dahliae strain JR2 and the corresponding regions in V. dahliae strain VdLs17 shown as dot plot. For clarity, only alignments with >95% sequence identity are displayed. (c) Magnification of *Cen3* of V. dahliae strain JR2 and the syntenic *Cen1* of strain VdLs17. Synteny between regions is indicated by ribbons; entire centromeric regions *Cen1* and *Cen3* are syntenic, and sequence similarity between individual *VdLTRE9* elements is visualized. The *Cen* regions ±150 kb are shown as well as genes (red), and repeats (blue) are annotated within this region. (d) Distribution of different repeat families in centromeres (*Cen*; dark blue) and across the genome (non-*Cen*; light gray) for V. dahliae strains VdLs17 and CQ2. The enrichment of specific subfamilies at centromeres was assessed using a one-sided Fisher exact test. Significant enrichment (multiple-testing corrected *P* value < 0.01) is denoted with an asterisk. (e to g) Hi-C contact matrix showing interaction frequencies between genomic regions in Verticillium dahliae strains JR2 (e), VdLs17 (f), and CQ2 (g). Regions of high interchromosomal interaction frequencies are indicative of centromeres and are highlighted by arrowheads. Interaction frequencies are summarized in 50-kb bins along the genome.

To examine if *VdLTRE9* similarly occurs at centromeres in other V. dahliae strains, we made use of the complete genome assembly of V. dahliae strain VdLs17 (8, 32, 35). The evolution of V. dahliae is characterized by chromosomal rearrangements (8, 35) (Fig. 4a; Fig. S4a to c). Nevertheless, synteny analyses between V. dahliae strains JR2 and VdLs17 revealed large regions of colinearity between chromosomes and identified significant sequence and synteny conservation between the centromeres and their flanking regions (Fig. 4b and c; Fig. S4a), suggesting that centromeric sequences and their locations are conserved. We queried the genome of V. dahliae strain VdLs17 for the presence of *VdLTRE9* and identified a single region on each chromosome, collectively containing 186 of the 207 (90%) complete or partial matches of *VdLTRE9* in the genome (Fig. 4d) (one-sided Fisher’s exact test; multiple-testing corrected *P* value 3e−146). These *VdLTRE9*-rich regions are ∼150 kb in size, AT rich, gene poor, and repeat rich and share similarity to the previously identified CenH3-bound and *VdLTRE9*-enriched regions of V. dahliae strain JR2 (Fig. 4b and c; Fig. S4d), suggesting that these regions similarly represent the centromeres of V. dahliae strain VdLs17.

10.1128/mBio.01714-20.4 FIG S4 (a to c) Whole-genome alignments between the eight chromosomes of Verticillium dahliae strains JR2 and VdLs17 (32) (a), V. dahliae strains CQ2 and JR2 (32, 33) (b), and V. dahliae strains CQ2 and VdLs17 (32, 33) (c). (d and e) Schematic overview of the genome assemblies of Verticillium dahliae strains VdLs17 (d) and CQ2 (e). The individual lanes show the GC content, the gene (red) and repeat (blue) density (all summarized in 5-kb windows with 500-bp slide), and the location of the centromere-associated *VdLTRE9*. (f) Synteny analyses of the eight chromosomes of V. dahliae strains JR2 and CQ2. Schematic overview of the eight chromosomes of V. dahliae strain JR2 (left) and the corresponding syntenic regions in V. dahliae strains CQ2 (right). Centromeres are indicated by stars, and syntenic centromeres of V. dahliae strain CQ2 are colored according to *Cen1* to -*8* of V. dahliae strain JR2. Download FIG S4, JPG file, 1.7 MB. Copyright © 2020 Seidl et al. 2020 Seidl et al. This content is distributed under the terms of the Creative Commons Attribution 4.0 International license.

Centromeres of N. crassa and some other fungi colocalize within the nucleus (15, 52–56). This colocalization can be experimentally determined using chromosome conformation capture (Hi-C), which can identify centromeres by their increased interchromosomal contacts (56). To confirm that Hi-C can be used to identify centromeres in V. dahliae, we first applied Hi-C to V. dahliae strain JR2. As anticipated, we observed seven strong interchromosomal contacts for each of the eight chromosomes (Fig. 4e). Importantly, the interacting regions overlap the CenH3-bound regions that we identified as centromeres (Table S1b), demonstrating that centromeres in V. dahliae strain JR2 colocalize within the nucleus and supporting that Hi-C reliably identifies centromeres (52, 53). We then applied Hi-C to V. dahliae strain VdLs17 and similarly identified regions with strong interchromosomal contacts, one for each of the chromosomes (Fig. 4f). These regions overlap the *VdLTRE9*-enriched regions (Table S1b), suggesting that these represent functional centromeres in V. dahliae strain VdLs17.

The two V. dahliae strains JR2 and VdLs17 are closely related and differ only by ∼0.05% sequence diversity (8, 35). Thus, the conservation of *VdLTRE9* at centromeres could be driven by limited divergence between the two V. dahliae strains rather than representing a hallmark of V. dahliae centromeres. Therefore, we sought to determine centromeres in an additional V. dahliae strain with increased sequence diversity compared with V. dahliae strain JR2 or VdLs17, namely, strain CQ2, which displays ∼1.05% sequence diversity (33). We previously obtained a long-read-based genome assembly of this strain that encompasses 17 contigs (33). We generated Hi-C data for V. dahliae strain CQ2 and utilized intrachromosomal contacts to assign the contigs into eight pseudochromosomes, leaving ∼148-kb unplaced scaffolds (Fig. 4g, Fig. S4e, and Table S1c). We subsequently identified a single region with seven strong interchromosomal contacts for each pseudochromosome that is significantly enriched for *VdLTRE9* (one-sided Fisher’s exact test; multiple-testing corrected *P* value 3.4e−166) (Fig. 4d and g, Fig. S4e, and Table S1b). Synteny analyses between V. dahliae strains JR2 and CQ2 revealed that the eight *VdLTRE9*-rich regions and their flanking chromosomal regions are colinear, suggesting that centromere locations are conserved between different V. dahliae strains (Fig. 4; Fig. S4a to c and f). With an average size of 165 kb, the centromeres of V. dahliae strain CQ2 are similar in size to the 144-kb and 157-kb average sizes in V. dahliae strains VdLs17 and JR2, respectively (Table S1b). The sizes of the corresponding (i.e., homologous) centromeres vary between the different V. dahliae strains. Yet, the consistent cooccurrence of the *VdLTRE9*-rich regions with the interaction data obtained by Hi-C throughout a selection of V. dahliae strains demonstrates that *VdLTRE9* is a hallmark of V. dahliae centromeres.

### The evolution of *Verticillium* centromeres.

**FIG 5:**
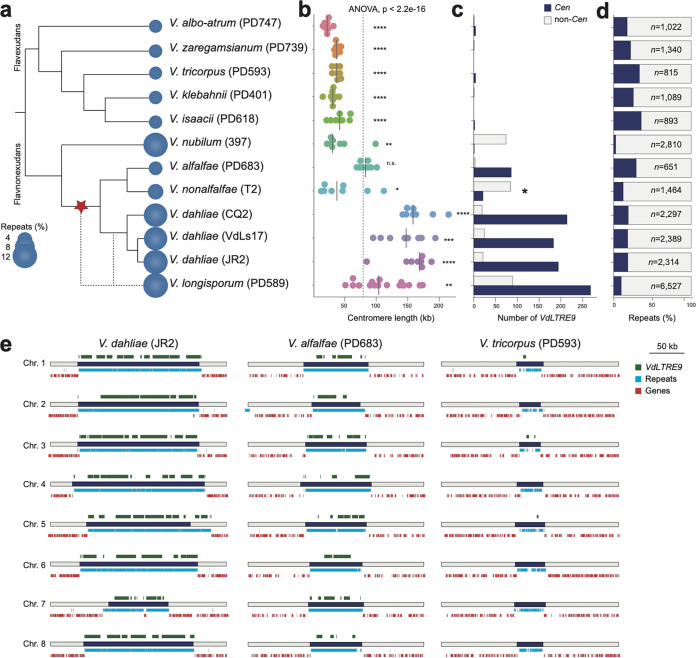
Evolution of centromeres in the genus *Verticillium.* (a) Relationship of the 10 members of the genus *Verticillium*. The predicted repeat content for each of the genomes is indicated (see Table S1c for details). The red star indicates the recruitment of *VdLTRE9* into centromeres. (b) Comparison of estimated centromere lengths (in kb) in the different *Verticillium* spp. Each dot represents a single centromere, and the line represents the median size. (c) The number of (partial) *VdLTRE9* matches identified in centromeres (*Cen*; dark blue) and across the genome (non-*Cen*; light gray). The asterisk indicates the high number of *VdLTRE9* elements in unassigned contigs for *Verticillium nonalfalfae* strain T2 (see the text for details). (d) Proportion of predicted repeat content localized at centromeres (*Cen*; dark blue) and across the genome (non-*Cen*; light gray). (e) Schematic overview of the eight centromeric regions (250 kb) in Verticillium dahliae strain JR2, and *Verticillium alfalfae* strain PD683 and *Verticillium tricorpus* strain PD593 as representatives for clade Flavnonexudans and clade Flavexudans, respectively. The centromeres are indicated by dark blue bars. The predicted genes (red) and repeats (light blue) are shown below each centromere, and locations of (partial) *VdLTRE9* matches (light green) are shown above each centromere. Global statistical differences for the centromere sizes were calculated using one-way analysis of variance (ANOVA), and differences for each species compared to the overall mean were computed using unpaired *t* tests; *P* values <0.0001, ****; *P* values <0.001, ***; *P* values <0.01, **; *P* values <0.05, *; n.s., not significant.

In addition to V. dahliae, we previously generated genome assemblies of the eight haploid *Verticillium* species and the allodiploid *V. longisporum* (39, 40) (Fig. 5a) that ranged from 12 to 684 scaffolds (Table S1c). These 10 *Verticillium* species have been traditionally separated over two distinct clades, Flavnonexudans and Flavexudans (Fig. 5a) (37). We generated Hi-C data to study the composition and evolution of centromeres in the different *Verticillium* species. By using intrachromosomal interaction signals, we assigned the vast majority of the previously assembled contigs into eight pseudochromosomes for each of the haploid *Verticillium* species and 16 pseudochromosomes for the diploid *V. longisporum*, leaving between 0.5 kb and 2,022 kb unassigned (Fig. S5; Table S1c). For most genome assemblies, the pseudochromosomes contain one or both telomeric repeats (Table S1c), and thus, we conclude that all *Verticillium* strains have eight chromosomes and that this number doubled in *V. longisporum*. Based on the interchromosomal Hi-C interaction signals, we identified a single region with high interchromosomal contacts for each of the pseudochromosomes (Fig. S5; Table S1d), indicating that these are the centromeres in the different *Verticillium* species. The average centromere size in *Verticillium* is ∼80 kb, yet we observed significant differences between the species (Fig. 5b; Fig. S6a and b). Centromeres within the Flavexudans clade are similarly sized and significantly smaller than the genus-wide average. In contrast, V. dahliae and *V. longisporum* centromeres are significantly larger.

10.1128/mBio.01714-20.5 FIG S5 Hi-C contact matrix showing the interaction frequencies between genomic regions in *V. nonalfalfae* (T2) (a), *V. alfalfae* (PD683) (b), the allodiploid *V. longisporum* (PD589) (c), *V. nubilum* (397) (d), *V. albo-atrum* (PD747) (e), *V. zaregamsianum* (PD739) (f), *V. tricorpus* (PD593) (g), *V. klebahnii* (PD401) (h), and *V. isaacii* (PD618) (i). Regions of high interchromosomal interaction frequencies are indicative of centromeres and are highlighted by arrowheads, and the blue line indicates boundaries between the pseudochromosomes. Download FIG S5, JPG file, 1.7 MB. Copyright © 2020 Seidl et al. 2020 Seidl et al. This content is distributed under the terms of the Creative Commons Attribution 4.0 International license.

10.1128/mBio.01714-20.6 FIG S6 (a and b) Comparison of normalized read coverage and corrected centromere lengths for *Verticillium* species for which short-read data are available. (a) Counts per million mapped reads (CPM) normalized read coverage was calculated for GC-biased corrected short-read libraries in 50-bp genomic windows, excluding regions containing assembly gaps (N’s). Genomic windows are summarized in boxplots (outliers not shown) by genomic location, centromeric regions (*Cen*, blue), and noncentromeric regions (non-*Cen*, gray). (b) Centromeric lengths inferred by Hi-C data were “corrected” based on the ratio of normalized read depth between centromeres and noncentromeric regions per chromosomes. Differences for each species compared to the overall mean were computed using unpaired *t* tests; *P* values <0.0001, ****; *P* values <0.001, ***; *P* values <0.01, **; *P* values <0.05, *. (c) The number of BLASTn matches of the *VdLTRE9* consensus element to the genomes of the *Verticillium* species separated by their genomic location, centromeric regions (*Cen*, blue), and noncentromeric regions (non-*Cen*, gray). The overall number of base pairs (bp) covered by the BLASTn matches in each genome sequence is indicated. The asterisk denotes the high number of *VdLTRE9* matches to unassigned, non-*Cen* regions in the genome assembly of *Verticillium nonalfalfae* (T2). (d) The number of repetitive element matches identified by RepeatMasker for each *Verticillium* species based on species/strain-specific repeat libraries generated by RepeatModeler separated by their genomic location, centromeric regions (*Cen*, blue), and noncentromeric regions (non-*Cen*, gray). (e) GC content of the *Verticillium* genomes in 50-bp windows and separated by their genomic location, centromeric regions (*Cen*, blue), and noncentromeric regions (non-*Cen*, gray). (f) The repeat content of centromeric regions in percent covered sequences in the different *Verticillium* species. Each data point summarized in the boxplot is the repeat content per centromere. Download FIG S6, JPG file, 2.8 MB. Copyright © 2020 Seidl et al. 2020 Seidl et al. This content is distributed under the terms of the Creative Commons Attribution 4.0 International license.

We subsequently assessed whether *VdLTRE9* defines centromeres in the other *Verticillium* species besides V. dahliae as well. Interestingly, *VdLTRE9* is abundant at centromeres in the allodiploid *V. longisporum* and in V. alfalfae, but fewer (21) or no *VdLTRE9* copies were identified at centromeres in *V. nonalfalfae* and *V. nubilum*, respectively (Fig. 5c and e; Fig. S6c and d). Similar to V. dahliae, the vast majority of matches are fragmented, suggesting that *VdLTRE9* has been significantly degenerated in these species as well. Only very few partial or no matches of *VdLTRE9* consensus could be identified in the genomes of the Flavexudans species (Fig. 5c and e; Fig. S6 and S7; Table S1e). Collectively, these findings suggest that *VdLTRE9* is specific to Flavnonexudans species, yet we cannot exclude the alternative scenario in which *VdLTRE9* was present at the last common ancestor of *Verticillium* and has been lost in all Flavexudans species. Regardless of the origin, *VdLTRE9* has likely been recruited to the centromeres of Flavnonexudans species only after the divergence of *V. nubilum* (Fig. 5a; Fig. S6 and S7).

10.1128/mBio.01714-20.7 FIG S7 Schematic overview of the centromeric regions (250 kb) in Verticillium dahliae strain JR2 (a), in species belonging to clade Flavnonexudans (b), and in species belonging to clade Flavexudans (c). The centromeres are indicated by dark gray bars. The predicted genes (black) and repeats (blue) are shown below each centromere, and locations of *VdLTRE9* (partial) matches (dark green) are shown above each centromere. Repeats that share sequence similarity (BLASTn) to the *VdLTRE9* consensus sequence are shown above each centromere (orange). Download FIG S7, JPG file, 0.6 MB. Copyright © 2020 Seidl et al. 2020 Seidl et al. This content is distributed under the terms of the Creative Commons Attribution 4.0 International license.

Since *VdLTRE9* occurs only in a few *Verticillium* species, we assessed to which extent other repetitive elements contribute to centromere organization. We analyzed the repeats identified by *de novo* repeat predictions for each of the *Verticillium* species. Centromeres in all species are AT and repeat rich (Fig. 5d and e; Fig. S6a and b), and some repeats occur in high frequency or nearly exclusively at centromeres in species that lack *VdLTRE9* (Table S1e). However, in contrast to *VdLTRE9*, these repeats cover only a minority (typically less than 10%) of the centromeres (Table S1e). Sequence similarity-based cluster analyses of the *de novo* repeat consensus sequences revealed that divergent repeat families contribute to *Verticillium* centromere organization (Fig. S8). Thus, in contrast to *VdLTRE9* in most Flavnonexudans species, we could not identify any additional repeat family as a hallmark of centromeres in other *Verticillium* species.

10.1128/mBio.01714-20.8 FIG S8 Sequence comparisons of *de novo* repeat families identified with RepeatModeler and RepeatMasker in the genome assemblies of the different *Verticillium* species. Individual repeat family consensus sequences were clustered using BLASTClust. (a) Relationships between different repeat family consensus sequences are displayed as connected graphs. The subgraph with the consensus sequences with similarity to *VdLTRE9* is highlighted in yellow. (b) The presence/absence matrix indicates the occurrences of different repeat families in the analyzed *Verticillium* species (black for present, white for absent). The cluster containing consensus sequences with similarity to *VdLTRE9* is highlighted. Download FIG S8, JPG file, 0.5 MB. Copyright © 2020 Seidl et al. 2020 Seidl et al. This content is distributed under the terms of the Creative Commons Attribution 4.0 International license.

### Centromeres contribute to *Verticillium* karyotype evolution.

**FIG 6:**
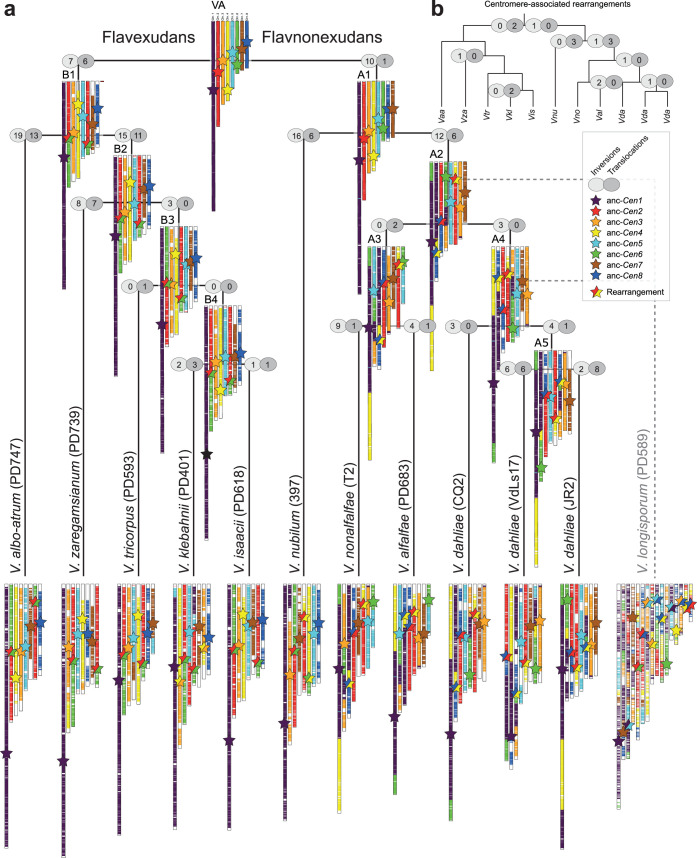
Centromeres contribute to karyotype evolution in *Verticillium.* (a) Relationship of the 10 members of the genus *Verticillium*. The allodiploidization event forming *V. longisporum* is indicated by dashed lines (38, 114). The chromosomal evolution within the haploid members of the genus was reconstructed using AnChro (57). The chromosomal structure of the nine species is shown in relation to the last common ancestor of the genus. The approximate locations of the centromeres are indicated by stars. The number of chromosomal rearrangements (inversions and translocations; see text) is displayed for each branch, and centromeres that colocalize in proximity to chromosomal rearrangements are highlighted by two-colored stars. (b) The number of major chromosomal rearrangements that occurred at, or in close proximity to, centromeres is shown along the branches depicting the *Verticillium* species phylogeny shown in panel a.

We previously used fragmented genome assemblies to identify chromosomal rearrangements during *Verticillium* evolution (8, 35, 40). We hypothesize that centromeres might have contributed to these chromosomal rearrangements. To identify genome rearrangements and to trace centromeres during *Verticillium* evolution, we used the pseudochromosomes of the haploid *Verticillium* species to reconstruct ancestral chromosomal configurations using AnChro (Fig. 6a) (57). We reconstructed all potential ancestors that predominantly had eight chromosomes and ∼8,000 genes (Fig. S9a and b), yet the number of ancestral chromosomes and genes varied when approaching the last common ancestor (Fig. S9a and b). By balancing the number of reconstructed chromosomes and genes, we identified a single most parsimonious ancestral genome with eight chromosomes and ∼8,500 genes (Fig. 6a; Fig. S9c), except for the last common ancestor within the Flavexudans clade that had eight major chromosomes and two additional “chromosomes” with only six and two genes (Fig. S9d). As these two smaller “chromosomes” likely do not represent genuine chromosomes, we conclude that all of the ancestral genomes, similarly to the extant haploid *Verticillium* genomes, had eight chromosomes (Fig. 6a). Confirming our previous report (40), we observed in total 198 chromosomal rearrangements (124 inversions and 74 translocations, including other complex rearrangements) (Fig. 6a). The number of chromosomal rearrangements is lower than previously recorded, and we did not observe any chromosomal fusion or fission events, which is likely the result of the drastically improved genome assemblies, but the rearrangement signal on each branch is sufficient to nevertheless recapitulate the known *Verticillium* species phylogeny (Fig. S9e). Importantly, we observed 17 genomic rearrangements that occurred at, or in close proximity (within ∼15 genes up- or downstream) to, centromeres, both in extant *Verticillium* species and in the ancestors (Fig. 6). For example, at the branch from the last common ancestor (VA, Fig. 6a) to the ancestor of the clade Flavexudans (B1, Fig. 6a), two centromere-associated translocations (between the ancestral chromosomes 2 and 6) led to the formation of two rearranged chromosomes. In total, we observed that five out of the eight ancestral centromeres were associated with a chromosomal rearrangement at one point during evolution (Fig. 6a). Nevertheless, comparisons of protein-coding genes that flank centromeres show that these are syntenic in most extant species. Similarly, none of the recent chromosomal rearrangements observed between V. dahliae strains is clearly associated with centromeres (Fig. 4a and b and Fig. 6a), even though *CEN2* of V. dahliae strain VdLs17 is located near (20 to 25 genes up-/downstream) a chromosomal rearrangement (Fig. 4a). Thus, while chromosomal rearrangements involving centromeres occurred during evolution, they do not account for the majority of the karyotype variation between extant *Verticillium* species.

10.1128/mBio.01714-20.9 FIG S9 Reconstruction of ancestral genomes within the genus *Verticillium* with AnChro (57). (a and b) The number of chromosomes (a) and genes (b) predicted by all potential ancestral reconstructions using different combinations of genomes and stringency parameters. The phylogenetic tree in panel a depicts the relationships between *Verticillium* species and the abbreviations used for the ancestors. The inlays display boxplots to summarize the number of chromosomes (a) and genes (b) per ancestral reconstruction. (c) The number of chromosomes and genes of the chosen “optimal” reconstruction for each of the internal ancestors. (d) The number of genes per chromosome for each of the reconstructed ancestor and the extant *Verticillium* species. The star highlights the reconstruction for the B1 ancestor that had 10 chromosomes, but with two chromosomes with six and two genes. (e) Reconstruction of the *Verticillium* species phylogeny based on synteny relationship using PhyChro (113). Download FIG S9, JPG file, 1.2 MB. Copyright © 2020 Seidl et al. 2020 Seidl et al. This content is distributed under the terms of the Creative Commons Attribution 4.0 International license.

## DISCUSSION

Centromeric regions are among the most rapidly evolving genomic regions (13–16, 29), yet centromere evolution has been systematically studied in only a few fungi (11, 12, 16, 29). Here, we took advantage of the fungal genus *Verticillium* and used a combination of genetic and genomic strategies to identify and characterize centromere organization and evolution. *Verticillium* centromeres are characterized as large regional centromeres that are repeat rich and embedded in heterochromatin. We furthermore show that centromeres contribute to the karyotype evolution of *Verticillium*. Finally, we demonstrate that *VdLTRE9* is a hallmark of centromeres in some *Verticillium* species, while species that lack *VdLTRE9* display a divergent repeat content.

Centromeres in fungi, plants, and animals colocalize within the nucleus (15, 52–56, 58), a phenomenon that can be exploited for their identification (52, 53). Here, we used Hi-C to first establish chromosome-level genome assemblies and subsequently identify centromeres in every *Verticillium* species, and we demonstrate that centromere locations are in agreement with CenH3 binding. While we obtained chromosome-level genome assemblies for all species, Hi-C scaffolded genome assemblies could still contain partially collapsed repeats and assembly gaps, in particular for short-read assemblies (59). With the exception of *V. nonalfalfae*, we observed only a few sequencing gaps and no evidence that would point to collapsed repeats at centromeres, suggesting that the inferred centromeres are of high quality. *Verticillium* centromere sizes differ, which is likely not driven by assembly artifacts, and centromeres in most *Verticillium* species are larger than in *Z. tritici* (27), C. neoformans, Magnaporthe oryzae, or Fusarium graminearum (13, 16, 29), yet smaller than in N. crassa (25). Species of the Flavexudans clade typically encode fewer repeats than species of the clade Flavnonexudans clade (32, 40, 60), and *V. nubilum*, *V. longisporum*, and V. dahliae are particularly rich in repeats compared with other *Verticillium* species (32, 39, 40, 42, 60). Thus, increased centromere sizes positively correlate with overall increased repeat contents.

Using fragmented genome assemblies, we previously identified chromosomal rearrangements during *Verticillium* evolution, which contributed to the formation of hypervariable LS regions containing genes with important roles in pathogen virulence (8, 35, 40). Thus, we proposed that chromosomal rearrangements in *Verticillium* contributed to genetic diversity and adaptation in the absence of sexual recombination (7, 35, 40). Chromosome-level genome assemblies for an entire genus enabled unprecedented analyses of the karyotype evolution over longer evolutionary timescales. Here, we observed extensive chromosomal rearrangements and provide evidence that some rearrangements at centromeres contributed to karyotype evolution, most of which occurred early during the divergence of *Verticillium*. Chromosomal rearrangements at centromeres occur in the yeasts *Candida*, *Cryptococcus*, and *Malassezia* (11, 12, 41), and synteny breakpoints have been identified between mammals and chicken (61), suggesting that centromeres often contribute to karyotype evolution. The emergence of chromosomal rearrangements at centromeres could be facilitated by their repeat-rich nature (11, 12). For example, centromeres in *Malassezia* are enriched with an AT-rich motif that could facilitate replication fork stalling, which leads to double-strand DNA breaks (11). Repeats localized outside centromeres in V. dahliae contribute to chromosomal rearrangements (8), and thus, it seems plausible that centromeric repeats similarly contribute to chromosomal rearrangements. It is tempting to speculate that the additional larger AT- and repeat-rich regions outside the centromeres (e.g., on chromosome 1, 7, or 8 of V. dahliae strain JR2) might have been involved in chromosomal rearrangements. However, based on our ancestral chromosome reconstruction, these regions, and even the entire chromosome (e.g., chromosome 8), are conserved and do not colocalize with any of the predicted large-scale translocations, even though smaller rearrangements might have occurred that have remained undetected. Chromosomal rearrangements often do not lead to changes only in chromosome organization but also in chromosome number (11, 12). While we observed chromosomal rearrangements, all extant and ancestral genomes contained eight chromosomes, suggesting that eight chromosomes are a stable configuration for all *Verticillium* species.

Centromere position and function are thought to be driven by the protein complement (e.g., CenH3 localization) and by heterochromatin formation rather than by specific DNA sequences (13, 15, 62). In V. dahliae, we observed the cooccurrence of CenH3 with H3K9me3 and DNA methylation. This suggests that DNA methylation, as previously reported in N. crassa and in C. neoformans (16, 25), is also a feature of centromeric DNA in V. dahliae. Colocalization of CenH3 with H3K9me2/3 and DNA methylation has been reported for N. crassa (25) and C. neoformans (16). In contrast, H3K9me3 and H3K27me3 are absent from centromeres in *Z. tritici* (27). H3K4me2 borders most centromeres in *Z. tritici* (27) and is associated with centromeres in Schizosaccharomyces pombe and some animals and plants (63–66). H3K4me2 has not been observed at centromeres in most fungi, including V. dahliae, and in the oomycete plant pathogen Phytophthora sojae (30). Changes in heterochromatin in N. crassa lead to altered CenH3 positioning (25), suggesting that heterochromatin is similarly required for centromere maintenance and function in V. dahliae. Elevated AT levels in repeat-rich heterochromatic regions can be caused by RIP mutations (15, 25, 26, 43). RIP-like mutations have been previously reported in some repeats in V. dahliae (36, 45), and we observed strong RIP signals at centromeres. Due to its presumably asexual nature (7), the occurrence of RIP in V. dahliae is controversial (8, 44, 45). Noteworthy, mutational signatures resembling RIP have recently been observed in *Z. tritici* propagated through mitotic cell divisions, pointing to the existence of a mitotic version of a RIP-like process (43). Thus, we conclude that RIP was an active process in V. dahliae at some point in evolution, or that RIP-like processes outside the sexual cycle occur in V. dahliae. Furthermore, a mechanistic link between AT-rich RIP mutated DNA, H3K9me3 deposition, and DNA methylation has been established in N. crassa (67), suggesting that these processes are also connected in V. dahliae.

Centromeres are often enriched for a variety of different retrotransposons and other repetitive elements (15, 16, 25, 29, 30, 68–70). We similarly observed that centromeres in all *Verticillium* species are repeat rich. Repeats and their remnants identified at centromeres typically also occur outside centromeres, as observed in *M. oryzae* (29) and N. crassa (25), for instance. Strikingly, we observed that a single degenerated LTR retrotransposon, *VdLTRE9*, is strongly associated with centromeres in some *Verticillium* species, while it is absent from LS regions in V. dahliae. The association of specific retrotransposons with centromeres has also been observed in the yeasts Ogataea polymorpha (69), Debaryomyces hansenii (68), and Scheffersomyces stipitis (70), where a retrotransposon related to *Ty5* is enriched at centromeres. Similarly, centromeres in *Cryptococcus* contain six retrotransposons (*Tcn1* to -*6*) that occur nearly exclusively at centromeres (16). Centromeres of *P. sojae* contain multiple types of repeats, but they are enriched for a single element called CoLT (*Copia*-like transposon) (30). The strong associations of specific repeats with centromeres could directly or indirectly link these elements to centromere function. Functional centromeres as observed here are also heterochromatic and contain CenH3. AT-rich repetitive elements can direct heterochromatin formation via DNA methylation and H3K9me3 deposition in N. crassa (46, 67), a phenomenon that can also occur at repeats outside centromeres (46). Heterochromatin occurs at centromeres but also at repeat-rich regions outside centromeres in V. dahliae; thus, the repeat-rich nature of centromeres is likely not sufficient to direct CenH3 deposition. In S. pombe heterochromatin formation is directed by short interfering RNAs (siRNAs) derived from flanking repetitive elements via RNAi (71, 72), and RNAi and heterochromatin mediate CenH3 localization at centromeres (73, 74). RNAi is also important for centromere maintenance and evolution in *Cryptococcus*, as RNAi-deficient species have smaller centromeres than RNAi-proficient ones (16). Interestingly, centromere-specific elements (*Tcn1* to -*6*) in RNAi-proficient species are typically full-length elements while only remnants can be found in RNAi-deficient species, which could be caused by recombination between elements (16). Furthermore, the genome size of RNAi-deficient species is smaller than that of RNAi-proficient ones, and centromere size reduction is at least partially responsible for genome size differences (16). In *Verticillium*, centromere size differences correlate with an increase in repeat content and the recruitment of *VdLTRE9*, which is highly fragmented and likely nonactive. Genome size differences exist in haploid *Verticillium* (33 Mb to 36 Mb; see Table S1c in the supplemental material), yet these do not seem to correlate with centromere sizes. Even though key components of the RNAi machinery exist in all *Verticillium* species (75) (Table S1f), we know only little about their biological functions. Similarly to C. neoformans, we observed no transcriptional activity of *VdLTRE9* or any other repeat at centromeres, but it is unclear if this silencing is mediated by RNAi, is a consequence of their heterochromatic nature, is due to their fragmentation, or is a combination of these. Ultimately, unraveling how specific elements contribute to centromere identity necessitates future experiments. *VdLTRE9* occurs only in some *Verticillium* species and has likely been recruited to centromeres subsequent to the divergence of *V. nubilum*. Conversely, these observations raise further questions on the roles of repeats and mechanisms of centromeric identity in species without *VdLTRE9*. Repeats drive the formation of chromosomal rearrangements, which are crucial for the formation and maintenance of LS regions, and thus are important drivers of *Verticillium* genome evolution and function (8, 36). Here, we highlight their contributions to centromere diversity within the fungal genus *Verticillium* and demonstrate that also centromeres contributed to chromosomal evolution. Our analyses provide the framework for future research into the diversity or convergence of mechanisms establishing centromere identity and functioning, and to elucidate roles of centromeres in generating genomic diversity in fungi.

## MATERIALS AND METHODS

### Construction of Verticillium dahliae transformants expressing FLAG-tagged CenH3.

CenH3 and H3 homologs were identified in the predicted proteomes of V. dahliae strain JR2 (32) and selected other fungi through a BLAST sequence similarity search (blastp v2.9.0+; default settings, E value cutoff 1e−20) (76, 77) using the N. crassa CenH3 (Q7RXR3) and H3 (P07041) sequences as queries. Missing homologs of CenH3 or H3 were identified using manual BLAST (tBLASTn v2.9.0+; default settings) (76, 77) and exonerate (v2.2.0; default settings) (78) searches against the genome sequences. Protein sequences of selected CenH3 and H3 proteins were aligned using mafft (v7.271; default settings, LINSi) (79), and poorly aligned regions in the alignment were removed using trimAl (v1.2; default settings) (80). A phylogenetic tree was inferred with maximum-likelihood methods implemented in IQ-tree (v1.6.11) (81), and robustness was assessed by 1,000 rapid bootstrap replicates.

To construct the N-terminally FLAG-tagged CenH3 strain of V. dahliae, a recombinant DNA fragment was constructed into the binary vector PRF-HU2 (82) or PRF-GU2 for homologous recombination. The CenH3 locus, from V. dahliae strain JR2, was amplified as 3 fragments with overlapping sequences (see Table S1g in the supplemental material). The 5′-most fragment containing the promoter was amplified using primers A+B, the open reading frame (ORF) with primers C+D, the Hyg promoter and ORF with primers E+F, and the 3′ end of the CenH3 locus with primers G+H. The four fragments were combined by overlap PCR using primers A+H and cloned into a PspOMI and SphI linearized vector using Gibson assembly. The vector construction was confirmed by Sanger sequencing. Vectors were transformed to *Verticillium* with *Agrobacterium-*mediated transformation (83). Correct homologous recombination and replacement at the *CenH3* locus were verified by PCR amplification using primers I+J (Fig. S1b and Table S1g). Correct translation of the recombinant protein was assessed using Western analyses with anti-FLAG antibody (Fig. S1c). Briefly, proteins were extracted from 5-day-old cultures grown in 100 ml potato dextrose broth at 22°C with continuous shaking at 120 rpm. Mycelium was collected by straining over a double layer of Miracloth and subsequently snap-frozen in liquid nitrogen and ground with a mortar and pestle using liquid nitrogen. Approximately 0.3 g of ground mycelium was resuspended in 600 μl protein extraction buffer (50 mM HEPES, pH 7.5, 150 mM NaCl, 1 mM EDTA, 1% glycerol, 0.02% NP-40, 2 mM phenylmethanesulfonyl fluoride [PMSF], 100 μM leupeptin, 1 μg/ml pepstatin), briefly vortexed, incubated on ice for 15 min, and centrifuged at 4°C at 8,000 × *g* for 3 min. The supernatant was collected by transferring 20 μl to a new tube to serve as the input control, and the remaining ∼500 μl was transferred to a fresh microcentrifuge tube with 15 μl of anti-FLAG M2 affinity gel (catalog number A2220; Sigma-Aldrich, St. Louis, MO, USA) and incubated while rotating at 4°C for 1 h. Samples were centrifuged at 5,000 × *g*, 4°C, for 3 min, after which the supernatant was discarded. Samples were washed with 500 μl of lysis buffer, and the centrifugation and washing were repeated three times. Protein was eluted from the resin by adding 15 μl of lysis buffer and 20 μl of 2× Laemmli loading buffer (4% SDS, 20% glycerol, 0.004% bromophenol blue, 125 mM Tris HCl, pH 6.8) and boiled at 95°C for 3 min. Protein samples were separated on a 12% polyacrylamide gel and subsequently transferred to polyvinylidene difluoride (PVDF) membranes, blocked in 5% bovine serum albumin (BSA), washed twice in Tris-buffered saline–Tween (TBST), and incubated with 1:3,500 anti-FLAG antibody (monoclonal anti-FLAG M2; Merck KGaA, Darmstadt, Germany).

### Chromatin immunoprecipitation followed by high-throughput sequencing (ChIP-seq).

For each V. dahliae genotype, one million spores were added to 100 ml potato dextrose broth and incubated for 7 days at 22°C with continuous shaking at 120 rpm. Mycelium was collected by straining over a double layer of Miracloth and subsequently snap-frozen in liquid nitrogen and ground with a mortar and pestle using liquid nitrogen. All ground material (0.5 to 1 g per sample) was resuspended in 4 ml ChIP lysis buffer (50 mM HEPES-KOH, pH 7.5, 140 mM NaCl, 1 mM EDTA, 1% Triton X-100, 0.1% sodium deoxycholate [NaDOC]) and Dounce homogenized 40 times in a 10-cm^3^ glass tube with a tightly fitting pestle on 800 power with an RZR50 homogenizer (Heidolph, Schwabach, Germany), followed by five rounds of 20-s sonication on ice with 40 s of resting in between rounds with a Soniprep 150 (MSE, London, United Kingdom). Samples were redistributed to 2-ml tubes and pelleted for 2 min at maximum speed in a tabletop centrifuge. Supernatants were pooled per sample in a 15-ml tube together with 25 μl anti-FLAG M2 magnetic beads (Sigma-Aldrich, St. Louis, MO, USA) and incubated overnight at 4°C and with continuous rotation. Beads were captured on a magnetic stand and washed with wash buffer (50 mM Tris HCl, pH 8, 1 mM EDTA, 1% Triton X-100, 100 mM NaCl), high-salt wash buffer (50 mM Tris HCl, pH 8, 1 mM EDTA, 1% Triton X-100, 350 mM NaCl), LiCl wash buffer (10 mM Tris HCl, pH 8, 1 mM EDTA, 0.5% Triton X-100, 250 mM LiCl), and TE buffer (10 mM Tris HCl, pH 8, 1 mM EDTA). Chromatin was eluted twice from beads by addition of 100 μl preheated TES buffer (100 mM Tris HCl, pH 8, 1% SDS, 10 mM EDTA, 50 mM NaCl) and 10 min incubation at 65°C. Proteinase K (10 mg/ml, 2 μl) was added and incubated at 65°C for 5 h, followed by chloroform extraction. DNA was precipitated by addition of 2 volumes 100% ethanol, 1/10 volume 3 M NaOAc, pH 5.2, and 1/200 volume 20 mg/ml glycogen, and overnight incubation at −20°C.

Sequencing libraries were prepared using the TruSeq ChIP library preparation kit (Illumina, San Diego, CA) according to the manufacturer’s instructions, but without gel purification and with use of the Velocity DNA polymerase (BioLine, Luckenwalde, Germany) for 12 cycles of amplification for the FLAG-CenH3. H3K4me2 ChIP was performed as described previously (36), using an anti-H3K4me2 antibody (catalog no. 39913; ActiveMotif, Carlsbad, CA, USA). Single-end (125-bp) sequencing was performed on the Illumina HiSeq2500 platform at KeyGene N.V. (Wageningen, the Netherlands).

### Chromatin confirmation capturing followed by high-throughput sequencing (Hi-C).

We determined the inter- and intrachromosomal contact frequencies using Hi-C in V. dahliae strains CQ2, JR2, and VdLs17, as well as in V. albo-atrum strain PD747, *V. alfalfae* strain PD683, V. isaacii strain PD618, V. klebahnii strain PD401, *V. longisporum* strain PD589, *V. nonalfalfae* strain T2, *V. nubilum* strain 397, V. tricorpus strain PD593, and V. zaregamsianum strain PD739. For each strain, one million spores were added to 400 ml Potato dextrose broth and incubated for 6 days at 22°C with continuous shaking at 120 rpm. Mycelium was collected by straining over double-layer Miracloth, and 300 mg (fresh weight) was used as input for generating Hi-C sequencing libraries with the Proximo Hi-C kit (Microbe) (Phase Genomics, Seattle, WA, USA), according to manufacturer’s instructions. Briefly, samples were first cross-linked for 15 min at room temperature. Cross-linked mycelium was treated with fungal cell lysis solution (10 mM beta-mercaptoethanol, 15 mg/ml Glucanex, dissolved in phosphate-buffered saline at pH 7.4) for 1 h at 30°C, followed by snap-freezing in liquid nitrogen and grinding with a plastic pestle to obtain a powder. The resulting material was further lysed using the lysis buffers provided with the Hi-C kit, and chromatin was collected by centrifugation. Next, chromatin was fragmented at 37°C for 1 h and proximity ligation was performed at room temperature for 4 h. Reverse cross-linking was performed overnight at 65°C. The resulting soluble DNA was purified and bound to streptavidin beads. Library preparation was then performed, followed by on-bead library amplification by PCR (72°C for 5 min; 98°C for 30 s; 15 cycles of 98°C for 10 s, 62°C for 20 s, and 72°C for 50 s). Libraries were cleaned up and eluted from the beads. Final yields were determined by quantification using a Qubit 2.0 fluorometer (Invitrogen). Hi-C sequencing libraries of V. dahliae strains CQ2, JR2, and VdLs17 were paired-end (2 × 125 bp) sequenced on the Illumina HiSeq2500 platform at KeyGene N.V. (Wageningen, the Netherlands). Hi-C sequencing libraries of the other *Verticillium* species were paired-end (2 × 150 bp) sequenced on the NextSeq500 platform at USEQ (Utrecht, the Netherlands).

### *In vitro* transcriptome profiling using RNA-seq.

RNA sequencing (RNA-seq) of *V. albo-atrum* strain PD747, *V. isaacii* strain PD618, *V. klebahnii* strain PD401, *V. longisporum* strain PD589, *V. nonalfalfae* strain T2, *V. nubilum* strain 397, *V. tricorpus* strain PD593, and *V. zaregamsianum* strain PD739 was performed as described previously (36). Single-end (50-bp) sequencing was performed on the BGISeq500 platform at BGI (BGI Hong Kong).

### Analyses of high-throughput sequencing data.

High-throughput sequencing libraries (Table S1a) have been analyzed as follows. Illumina reads were quality-filtered and trimmed using Trimmomatic (version 0.36) (84). Sequencing reads were trimmed and filtered by removing Illumina TruSeq sequencing adapters (settings seed mismatches 2, palindrome clip threshold 30, and simple clip threshold 10), removal of low-quality leading or trailing bases below quality 5 and 10, respectively, and 4-base sliding window trimming and cutting when average quality per base dropped below 15. Additionally, filtered and trimmed reads of <90 nt were removed from further analyses. Filtered and trimmed reads were mapped to the corresponding genome assembly with Bowtie2 (default settings) (85), and mapping files were converted to bam-format using SAMtools (v 1.8) (115). Genomic coverage was determined using deepTools (v3.4.1; bamCoverage) (87) by extending sequencing reads to 147 bp followed by RPGC normalization with a bin size of 1,000 bp and smoothening of 3,000 bp. To assess between sample variability, we used deepTools (v3.4.1, plotPCA) (87) to generate principal-component analyses. Furthermore, we employed deepTools (v3.4.1, multiBigwigSummary) (87) to summarize genomic coverages of values over genes, repetitive elements, and genomic windows (5-kb windows with 500-bp slide). Genomic regions enriched for FLAG-CenH3 were identified using MACS2 (v2.1.1) (broad peak option; broad cutoff 0.0025) (88).

To determine DNA (cytosine) methylation, we utilized sequencing data of bisulfite-treated genomic DNA previously generated for V. dahliae strain JR2 (36). Sequencing reads were mapped to the V. dahliae strain JR2 genome assembly as previously described (36). Subsequently, the number of reads supporting cytosine methylation in CG context was extracted, and weighted CG-methylation levels were calculated over genes, repetitive elements, and genomic windows (5-kb window size with 500-bp slide) (89); weighted CG methylation was defined as the sum of reads supporting cytosine methylations divided by the sum of all reads occurring at all CG sites in the respective regions. Sites with less than four reads were not considered.

To improve the genome assemblies of the *Verticillium* species, we mapped Hi-C sequencing reads to genome assemblies of V. dahliae strain CQ2, *V. albo-atrum* strain PD747, *V. alfalfae* strain PD683, *V. isaacii* strain PD618, *V. klebahnii* strain PD401, *V. longisporum* strain PD589, *V. nonalfalfae* strain T2, *V. nubilum* strain 397, *V. tricorpus* strain PD593, and *V. zaregamsianum* strain PD739 using Juicer (v1.6) with early-stage setting (90). The contact matrices generated by Juicer were used by the three-dimensional (3D) *de novo* assembly (3D-DNA) pipeline (91) (v180922) with a contig size threshold of 1,000 bp to eliminate misjoints in the previous assemblies and to generate improved assemblies. The genome assemblies were manually improved using Juicebox Assembly Tools (JBAT) (v1.11.08) (92), and improved genome assemblies were generated using the 3D-DNA postreview asm pipeline (91). Centromere locations were determined using a 1-kb-resolution contact matrix in JBAT, by identifying a region per chromosome that displays strong interchromosomal interactions, yet weak intrachromosomal interactions (see Fig. S5).

To assess potential repeat collapses during genome assemblies at centromeric regions, we mapped previously generated short-read data for V. dahliae strains JR2 and VdLs17, *V. albo-atrum* strain PD747, *V. alfalfae* strain PD683, *V. isaacii* strain PD618, *V. klebahnii* strain PD401, *V. longisporum* strain PD589, *V. nonalfalfae* strain T2, *V. tricorpus* strain PD593, and *V. zaregamsianum* strain PD739 (34, 39, 40, 93) to the genome assemblies using BWA (v0.7.17; mem) (86). We first used bedtools (v2.29.2) (94) to identify genomic regions with >500× coverage. We then applied deepTools (v3.4.1, computeGCBias) (87) to compute GC biases of read depth across the genome, excluding the identified high-coverage regions, and used deepTools (v3.4.1, correctGCBias) (87) to correct GC biases, which addresses known biases in sequencing library preparation to ensure even read coverage throughout the genome irrespective of their base composition (95). We used deepTools (v3.4.1, bamCoverage, bins 50 bp, counts per million [CPM] normalization) (87) to obtain the read coverage throughout the genome, excluding regions containing sequence assembly gaps (N’s). Assuming that collapsed repeats would lead to a local increase in read depth, we used the ratio of the average read coverage at the centromeres and outside the centromere at each chromosome to correct the inferred centromere sizes. To further validate the genome assembly of regions identified as centromeres of V. dahliae strain JR2, the genome assembly was compared to the previously generated optical map (35) using MapSolver (v 3.2; OpGen, Gaithersburg, MD).

The transcriptional activity for genes and repetitive elements in V. dahliae strain JR2 was assessed *in vitro* (in potato dextrose broth) using previously generated deep transcriptome data sets (36). To this end, single-end sequencing reads of three biological replicates were mapped to the V. dahliae strain JR2 genome assembly (32) using STAR (v2.4.2a; maximum intron size 1 kb and outFilterMismatchNmax to 5) (96). The resulting mapped reads were summarized per genomic feature (gene or repeat) using summarizeOverlaps (97), converted to counts per million (CPM) mapped reads, and averaged over the three biological replicates.

### Sequence analyses of *Verticillium* genome assemblies, centromeres, and repeat and gene content.

Repetitive elements in the genomes of V. dahliae strains JR2, VdLs17, and CQ2 (32, 33) were identified as previously described (36). Briefly, repetitive elements were identified in each genome independently using a combination of LTRharvest (98) and LTRdigest (99) followed by identification of RepeatModeler. Identified repeats in the different V. dahliae strains were clustered into a nonredundant library that contained consensus sequences for each repeat family. The repeat library was, if possible, manually curated and annotated using PASTEC (100) or by sequence similarity to previously identified and characterized repeat families (32, 45). Genome-wide occurrences of repeat families were determined using RepeatMasker (v 4.0.9; sensitive option and cutoff 250), and the output was postprocessed using ‘One code to find them all’ (101). We considered only matches to the repeat consensus library and thereby excluded simple repeats and low-complexity regions.

*De novo* gene and repeat annotation for the Hi-C-improved *Verticillium* genome assemblies, and for V. dahliae strains JR2 and VdLs17 as a comparison, was performed using the funannotate pipeline (102). Briefly, repetitive elements were first *de novo* identified using RepeatModeler and masked for gene prediction using RepeatMasker. Subsequently, gene prediction parameters were estimated using *in vitro* RNA-seq data (see above for details; exceptions: *V. alfalfae*, for which no RNA-seq data were available; *V. nonalfalfae*, for which publicly available RNA-seq data were used [93]; and V. dahliae strain JR2, for which, in addition to the *in vitro* RNA-seq data generated in this study, also previously generated *in vitro* data [xylem sap and half-strength Murashige and Skoog {36}] as well as long-read nanopore cDNA data [103] were used). Based on the gene prediction parameters, gene prediction was performed with funannotate using a combination of *ab initio* gene predictors, consensus predictions were obtained using Evidencemodeler (v1.1.1) (104), and gene predictions were adjusted using information from the RNA-seq data. Repeat annotation for each genome assembly was based on the *de novo* repeat family consensus sequences obtained with funannotate. Genome-wide occurrences of these repeat families as well as previously defined repeat families for V. dahliae (see above) were determined using RepeatMasker (v 4.0.9; sensitive option and cutoff 250), and the output was postprocessed using ‘One code to find them all’ (101). *De novo* repeat families overlapping with centromeres in the different species were clustered using BLASTClust (v2.2.26; parameter ‘-S 60 -L 0.55 -b F -p F’) and subsequently visualized using Cytoscape (v.3.8.0) (105). Next to RepeatMasker, genome-wide occurrences of the previously determined *VdLTRE9* (32, 36) were identified by BLAST searches (blastn v2.9.0+; E value cutoff 1e−5, no soft-masking and dust, fixed database size 10e6) (76, 77), and similarity between VdLTRE9 consensus sequences and the *de novo* predicted repeat families was established using BLAST (blastn, E value cutoff 1e−5, query coverage >50%, no soft-masking and dust, fixed database size 10e6).

Repeat and gene density (V. dahliae strain JR2 and VdLs17 based on previous gene annotation [103]), GC content, and composite RIP index (CRI) were calculated along the genome sequence using sliding windows (5-kb window with 500-bp slide). The CRI was calculated according to the method of Lewis et al. (46). CRI was determined by subtracting the RIP substrate from the RIP product index, which are defined by dinucleotide frequencies as follows: RIP product index = TpA/ApT and the RIP substrate index = (CpA + TpG)/(ApC + GpT). Overlaps between different genomic features (for example, repetitive elements over centromeric regions) were assessed using bedtools (v2.29.2) (94). Genome-wide data were visualized using R (106) with the package ggplot2 (107), karyplotR (108), or Gviz (109), as well as EasyFig (110).

Whole-genome alignments between V. dahliae strains JR2, VdLs17, and CQ2 were performed using NUCmer, which is part of the MUMmer package (v 3.1; –maxmatch) (111). To remove short matches, we considered only alignments longer than 10 kb. Ancestral genome configurations were reconstructed using AnChro (56). We first determined the synteny relationships between all possible pairs of haploid *Verticillium* genomes and two outgroup genomes (Plectosphaerella cucumerina and Sodiomyces alkalinus) using SynChro with synteny block stringency (delta parameter) ranging from 2 to 5 (112). We then obtained all ancestors by calculating all possible pairs of genomes (G1 and G2) and outgroups (G3, …, G*_n_*) and by varying the delta′ (G1 and G2 comparisons) and deltaʺ (G1/G3...G1/G*_n_* and G2/G3...G2/G*_n_* comparisons) parameters for AnChro. We additionally reconstructed all ancestors starting from the extant genomes in a sequential approach with multiple successive cycles of SynChro and AnChro (delta parameters varied between 2 and 5). For each ancestor, we chose the optimal reconstruction by the combination of delta parameters (delta′ and deltaʺ) that minimizes the number of reconstructed chromosomes and rearrangements and at the same time maximizes the number of genes, both guided by the most commonly observed number of chromosomes and genes in all rearrangements. We obtained the number of large-scale rearrangements between reconstructed ancestral genomes and the extant *Verticillium* genomes using ReChro with a delta parameter of 1 (56). The relationship between chromosomes of the reconstructed ancestors and the extant species in relationship to the common ancestor is generated with SynChro with a delta parameter of 1 (112). A species phylogeny that uses synteny relationships computed by SynChro (see above) as informative character between the *Verticillium* genomes and the outgroup genomes was reconstructed using PhyChro (113).

### Data availability.

ChIP-seq and Hi-C data were submitted to the Short Read Archive (SRA) under the accession no. PRJNA641329 (Table S1a).
