# Supplementary figures and images for: Repetitive Elements Contribute to the Diversity and Evolution of Centromeres in the Fungal Genus Verticillium
Source: mBio. 2020 Sep 8;11(5):e01714-20. doi: 10.1128/mBio.01714-20 (PMC7482064; doi:10.1128/mBio.01714-20)

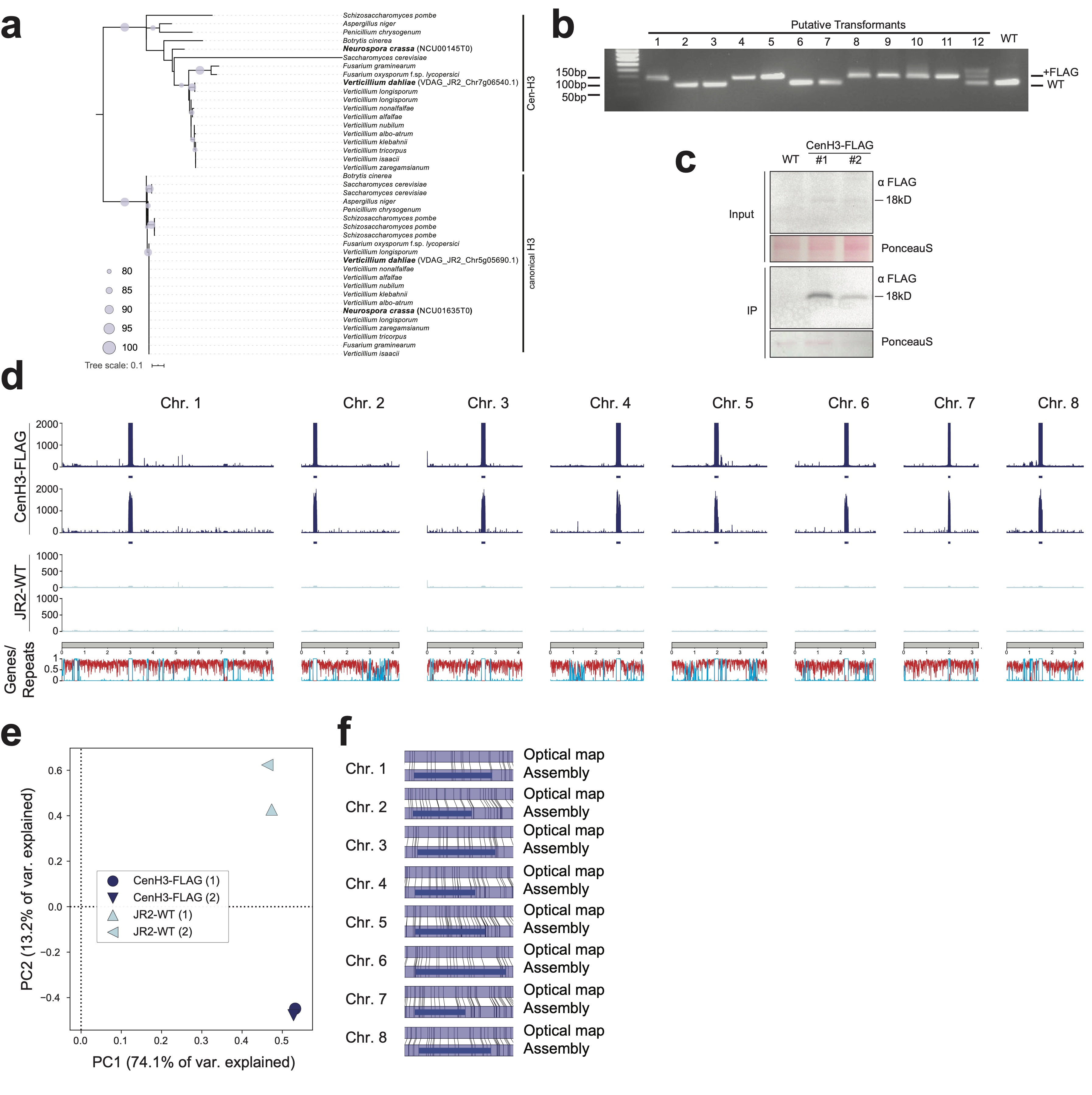

Supplement: FIG S1 [file mBio.01714-20-sf001.jpg]

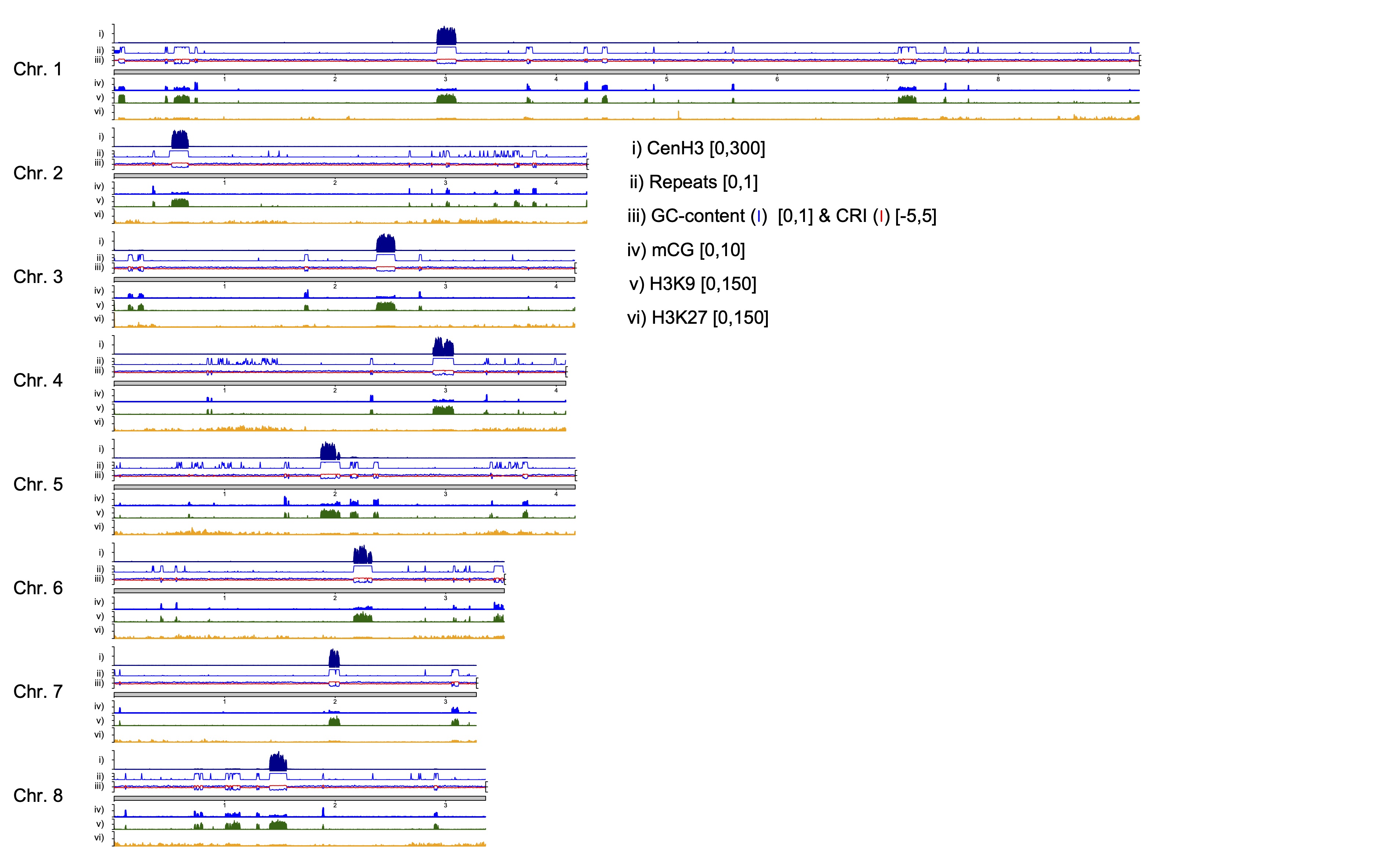

Supplement: FIG S2 [file mBio.01714-20-sf002.jpg]

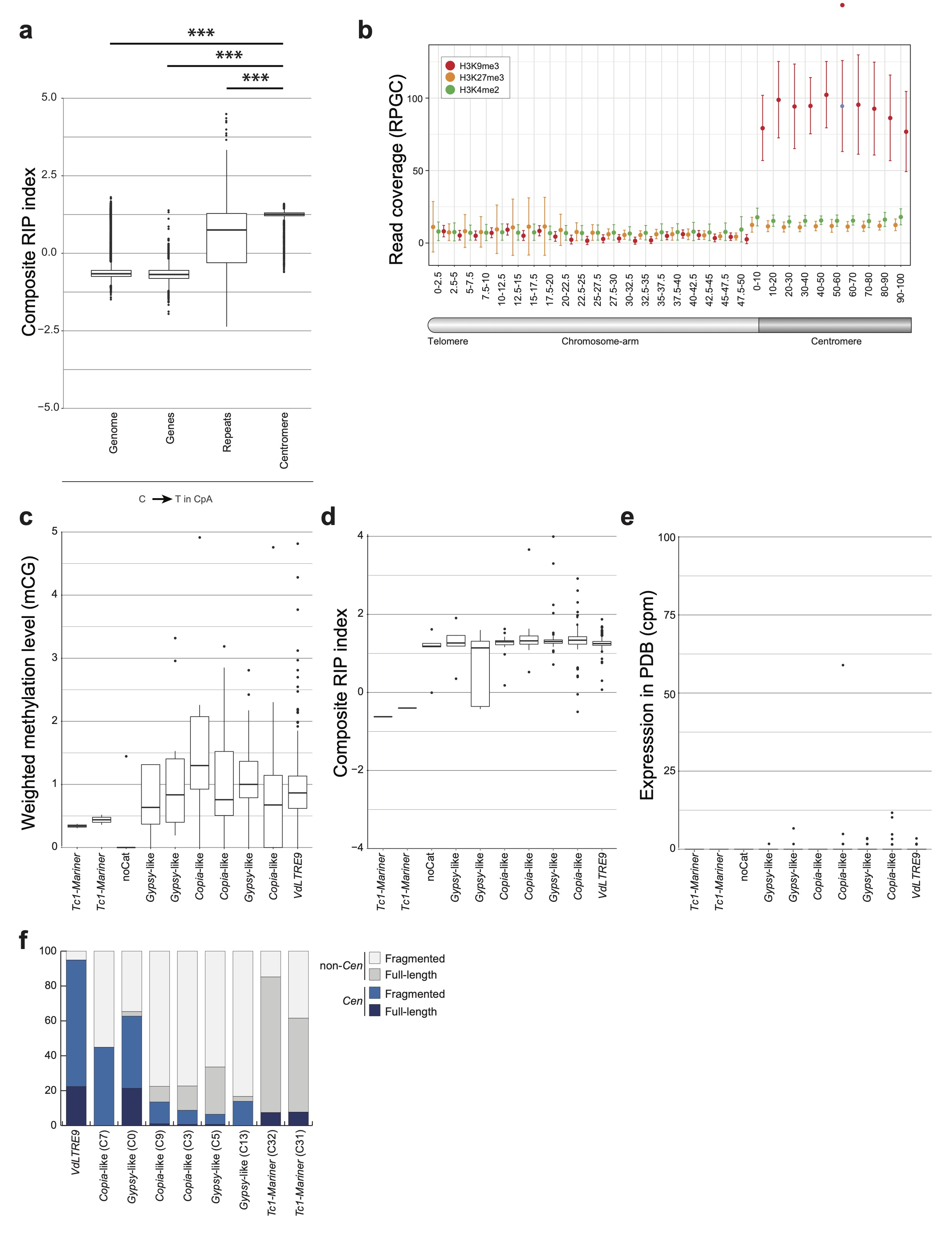

Supplement: FIG S3 [file mBio.01714-20-sf003.jpg]

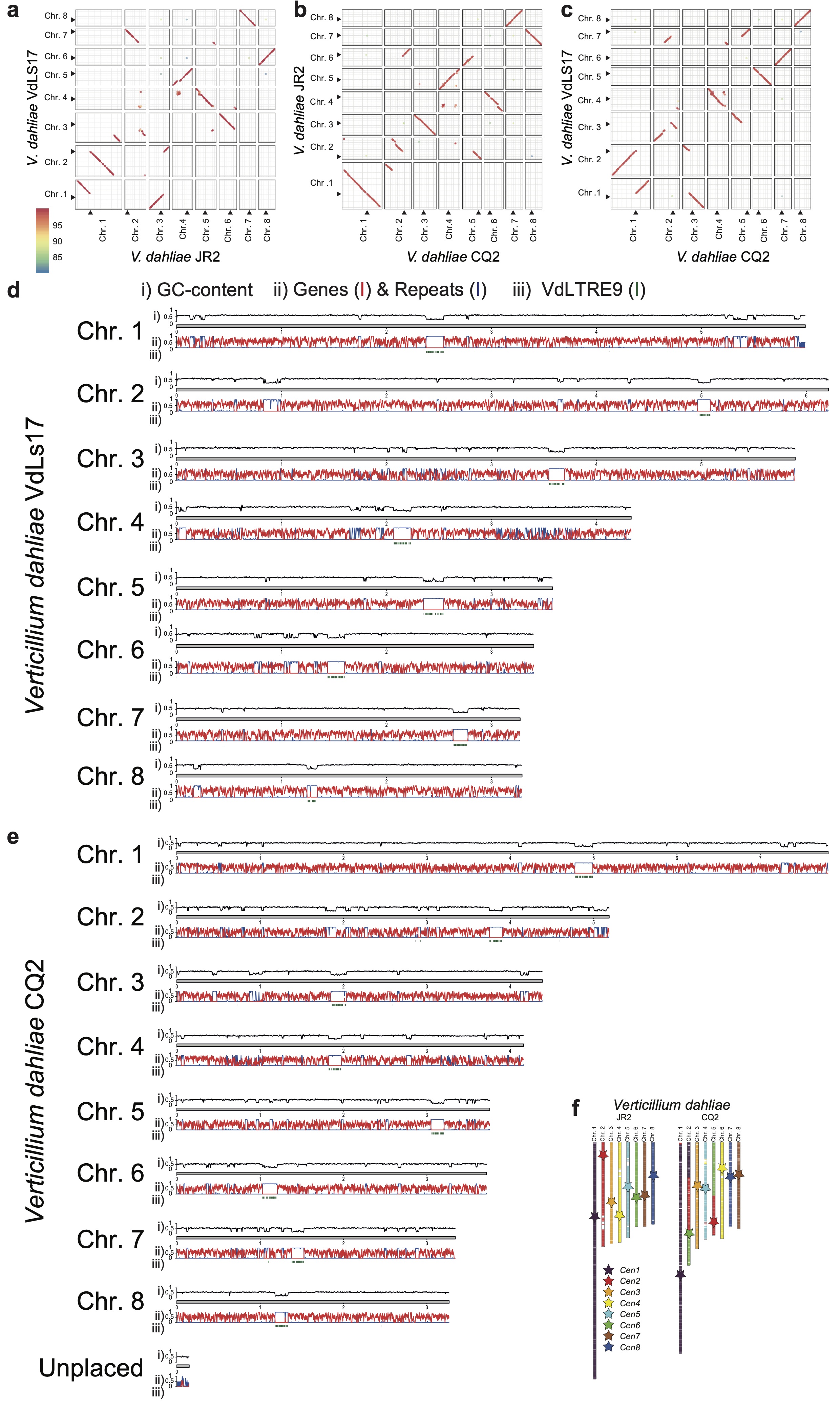

Supplement: FIG S4 [file mBio.01714-20-sf004.jpg]

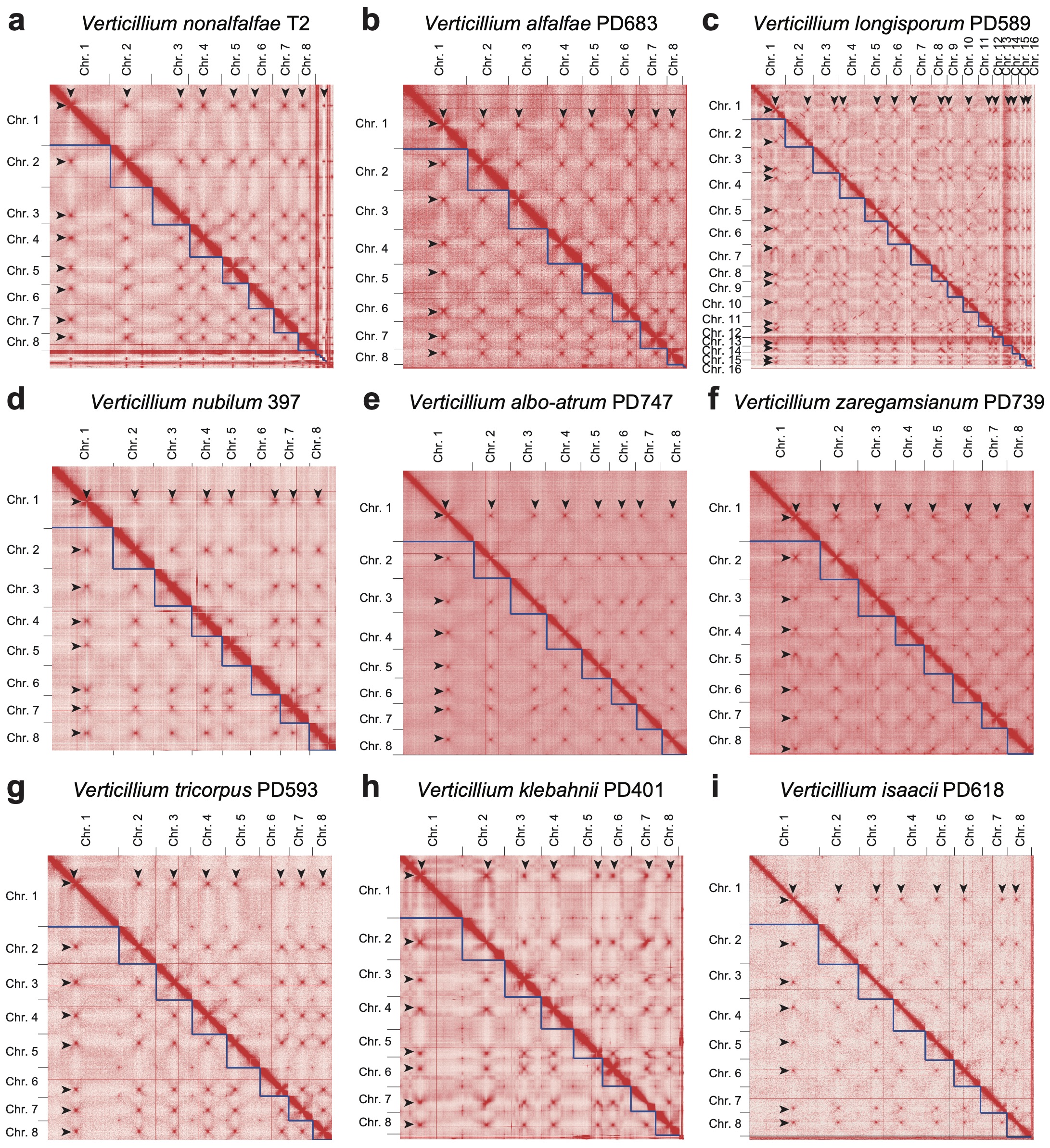

Supplement: FIG S5 [file mBio.01714-20-sf005.jpg]

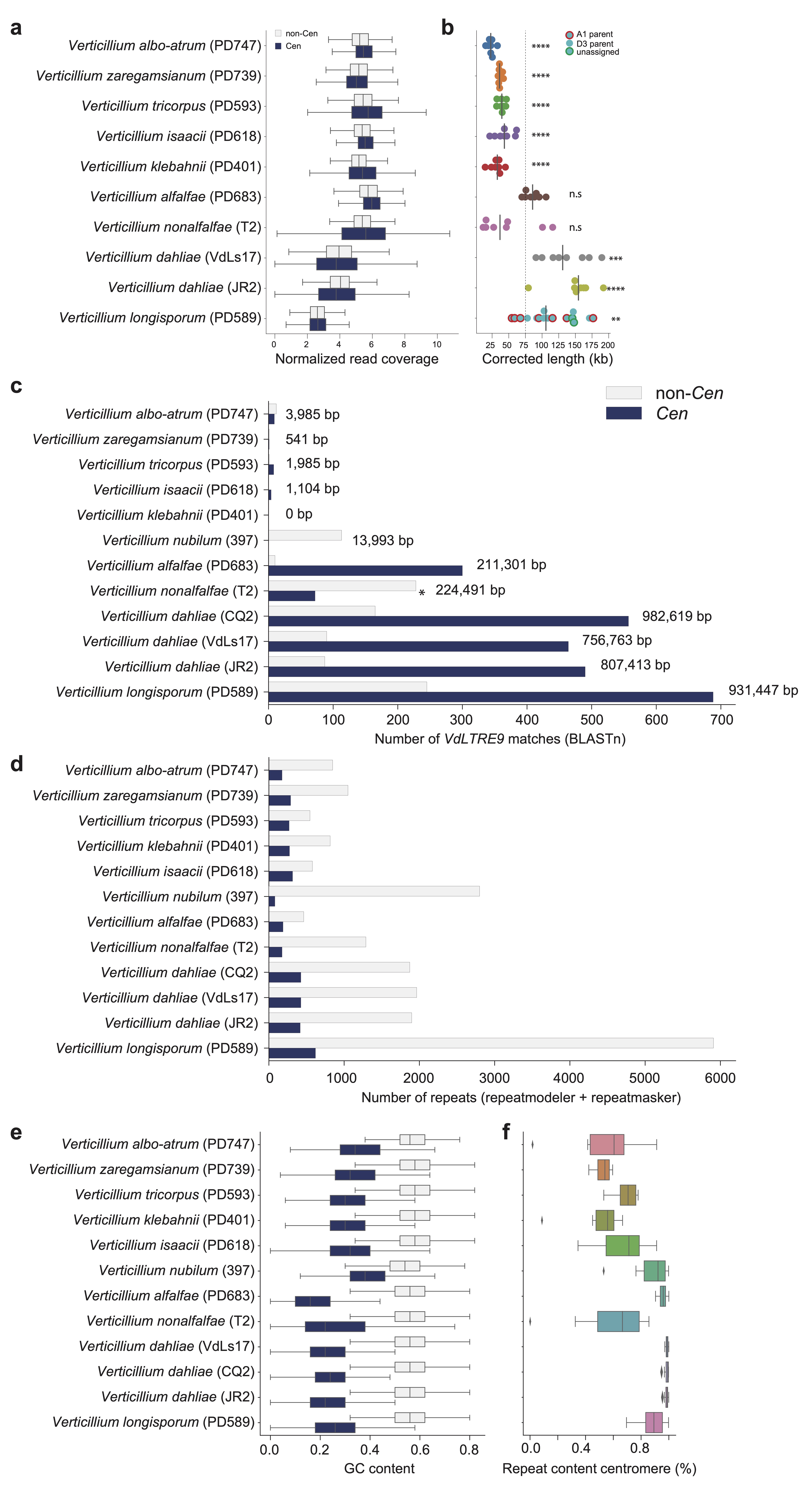

Supplement: FIG S6 [file mBio.01714-20-sf006.jpg]

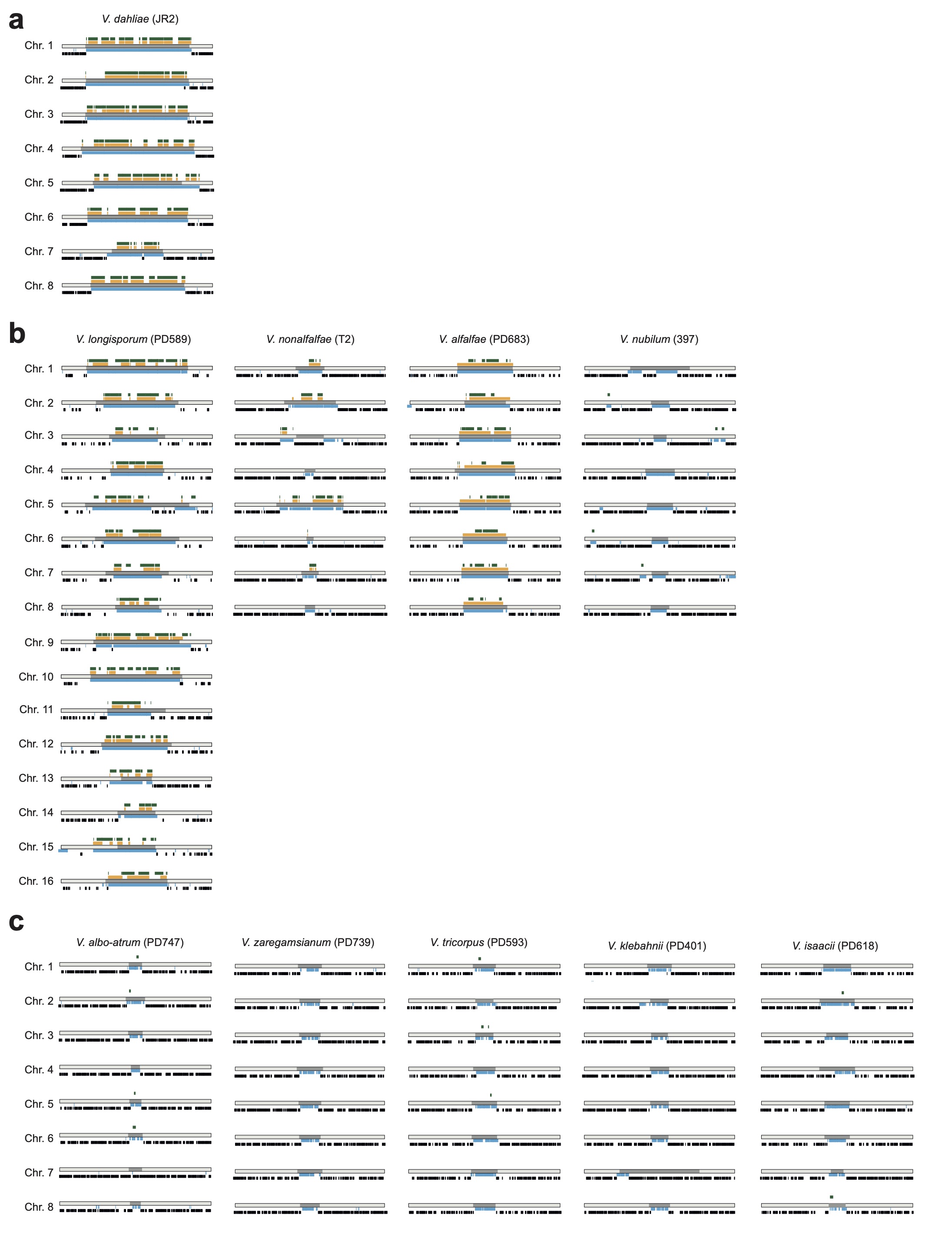

Supplement: FIG S7 [file mBio.01714-20-sf007.jpg]

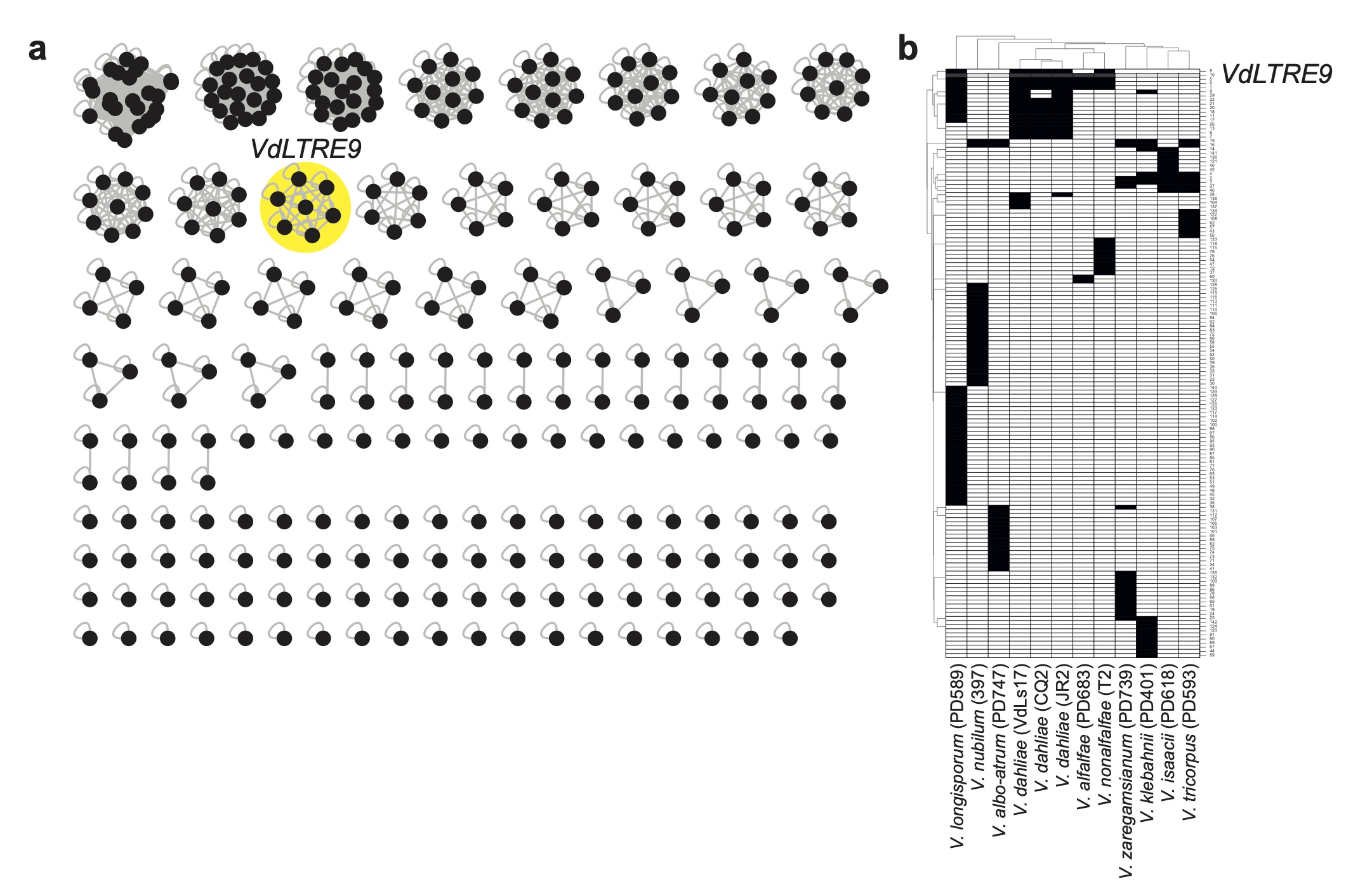

Supplement: FIG S8 [file mBio.01714-20-sf008.jpg]

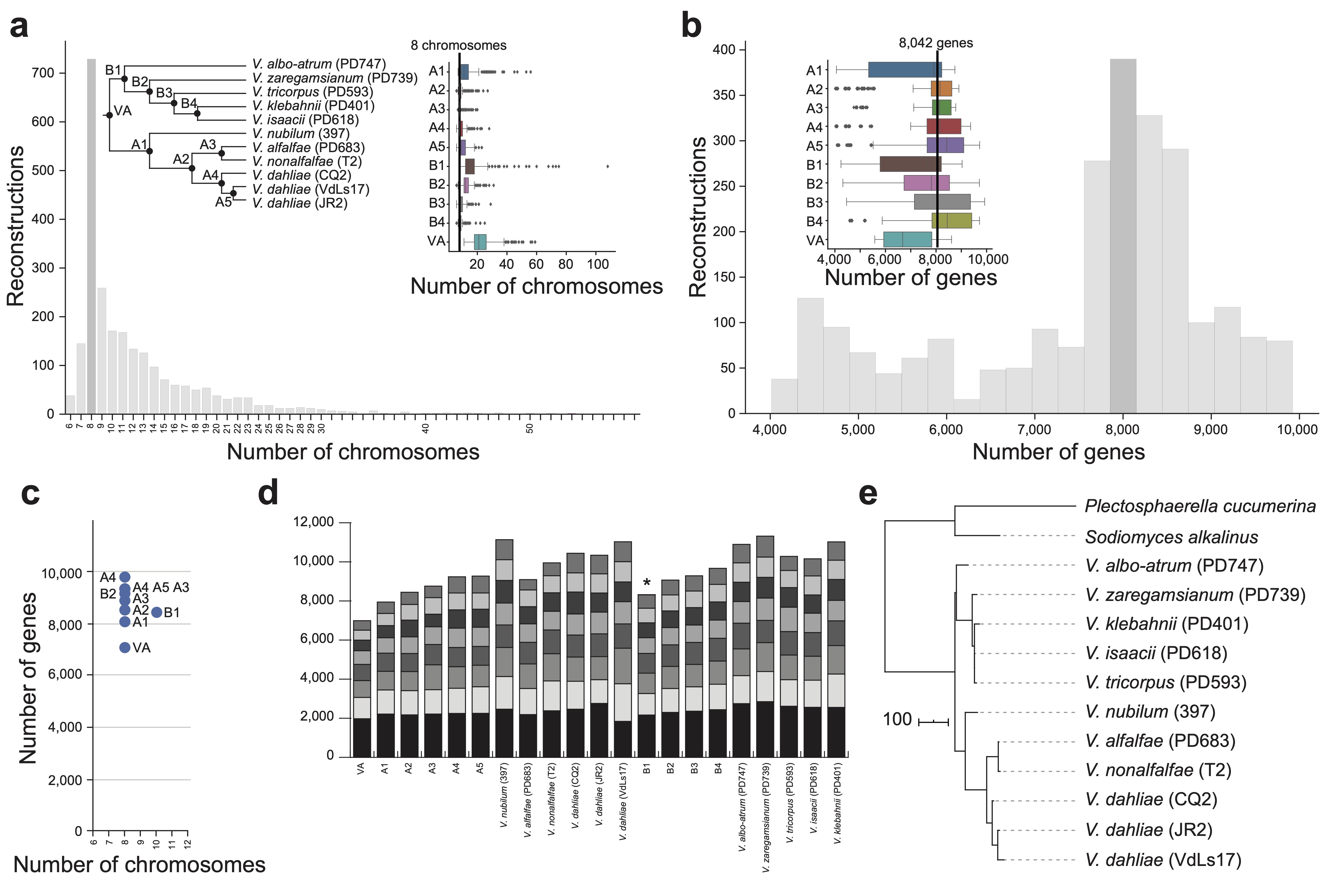

Supplement: FIG S9 [file mBio.01714-20-sf009.jpg]
